# Systematic Identification of Combinatorial Drivers and Targets in Cancer Cell Lines

**DOI:** 10.1371/journal.pone.0060339

**Published:** 2013-04-05

**Authors:** Adel Tabchy, Nevine Eltonsy, David E. Housman, Gordon B. Mills

**Affiliations:** 1 Department of Systems Biology, The University of Texas M. D. Anderson Cancer Center, Houston, Texas, United States of America; 2 Kleberg Center for Molecular Markers, The University of Texas M. D. Anderson Cancer Center, Houston, Texas, United States of America; 3 Sheikh Zayed Bin Sultan Al Nahyan Institute for Personalized Cancer Therapy, The University of Texas M. D. Anderson Cancer Center, Houston, Texas, United States of America; 4 The David H. Koch Institute for Integrative Cancer Research at MIT, Massachusetts Institute of Technology, Cambridge, Massachusetts, United States of America; 5 Department of Biology, Massachusetts Institute of Technology, Cambridge, Massachusetts, United States of America; Center for Genomic Regulation, Spain

## Abstract

There is an urgent need to elicit and validate highly efficacious targets for combinatorial intervention from large scale ongoing molecular characterization efforts of tumors. We established an in silico bioinformatic platform in concert with a high throughput screening platform evaluating 37 novel targeted agents in 669 extensively characterized cancer cell lines reflecting the genomic and tissue-type diversity of human cancers, to systematically identify combinatorial biomarkers of response and co-actionable targets in cancer. Genomic biomarkers discovered in a 141 cell line training set were validated in an independent 359 cell line test set. We identified co-occurring and mutually exclusive genomic events that represent potential drivers and combinatorial targets in cancer. We demonstrate multiple cooperating genomic events that predict sensitivity to drug intervention independent of tumor lineage. The coupling of scalable in silico and biologic high throughput cancer cell line platforms for the identification of co-events in cancer delivers rational combinatorial targets for synthetic lethal approaches with a high potential to pre-empt the emergence of resistance.

## Introduction

A major emerging challenge in the wake of the tsunami of data generated by efforts to characterize tumors at the molecular level (e.g. The Cancer Genome Atlas [TCGA] (http://www.cancergenome.nih.gov) and International Cancer Genome Consortium [ICGC] (http://www.icgc.org)) is how to leverage the data and translate it into improved clinical outcomes, by identifying the molecular basis of cancer in individual patients and subsequently using these molecular lesions as targets for effective intervention. At the same time, the reduction in sequencing costs leading to the *democratization* of molecular testing is already resulting in many patients having their tumors typed at a molecular level. The tumor characterization efforts are no longer rate limiting; rather how to interpret and “act” on the data is now the major limiting factor. These challenges must be overcome before emerging technological advances in tumor characterization can deliver maximum clinical impact. A key step in the process is the identification of biomarkers that would predict response to treatment and the parsing of actionable driving molecular aberrations from noise. These challenges can be solved by implementing algorithms that help analyze the data, in parallel to establishing large scale humanized model systems for high throughput target discovery and validation that will also inform an accelerated drug development and clinical trial process. Robust predictive biomarkers for combinatorial molecular medicine are urgently needed to change the clinical trial landscape from the current state of low therapeutic efficacy in large clinical trials and unselected populations, to high efficacy small clinical trials enriched for target populations. This approach has the potential to make clinical trials smaller, faster, and cheaper, while increasing the benefits for individual patients.

Thus far single biomarkers driven interventions have had limited success in the clinic. Initial successes with targeted therapeutics in “oncogene-addicted” tumors [1]–[4] (e.g. Imatinib in CML; BRAF inhibitors in melanoma) have been tempered by the realization of a series of limitations: (1) emergence of resistance due to cancer heterogeneity, with pre-existing clones demonstrating variation in the molecular target leading to clinical resistance (clonal selection); (2) initial resistance of tumors due to co-mutation in a resistance pathway; and (3) resistance due to homeostatic feedback loops that re-instate the baseline steady state perturbed by the targeted intervention [2]–[6]. Thus, it appears that single biomarkers and/or interventions may have limited potential for success in the clinic. In the same way that we manage life threatening bacterial or viral infections (e.g. Tuberculosis, Human Immunodeficiency Virus) with multiple simultaneous antibiotics [7]–[9], successful therapy for cancer, which has all the versatility and robustness of the eukaryotic repertoire of responses at its disposal, will most likely require multiple simultaneous targeted interventions to preempt the emergence of resistance. Here we propose a framework for the rational identification of the multiple drivers that cooperate to produce the cancer phenotype, and could then be used as effective targets for combined therapeutic intervention.

Cancer cell lines closely recapitulate known tumor-associated genetic abnormalities providing models for human disease. For instance, breast cancer-derived cell lines have been shown to faithfully recapitulate the genomic features of primary tumors, with HER2 gene amplification correlating with trastuzumab sensitivity both in vitro and in patients [10], demonstrating that clinically observed genotype-response correlations are conserved in cancer cell line models. Here we perform a systematic search for genomic co-events that are selected during cancer initiation or progression, and if targeted together could markedly improve patient outcomes. We demonstrate an in silico platform for the identification of co-occurring cancer drivers and biomarkers of response, and its application as proof of concept in a highly characterized 669 cell line set treated with 37 novel targeted agents. Predictive biomarkers identified in a 141 cell line training set were validated in an independent 359 cell line test set. We propose that a pipeline composed of a robust in silico bioinformatic platform coupled to a high throughput cancer cell line platform for functional genomic discovery and validation could act as a bridge between characterization efforts like the TCGA/ICGC on one end and the clinic/clinical trials on the other.

## Methods Summary

**Figure 1:**
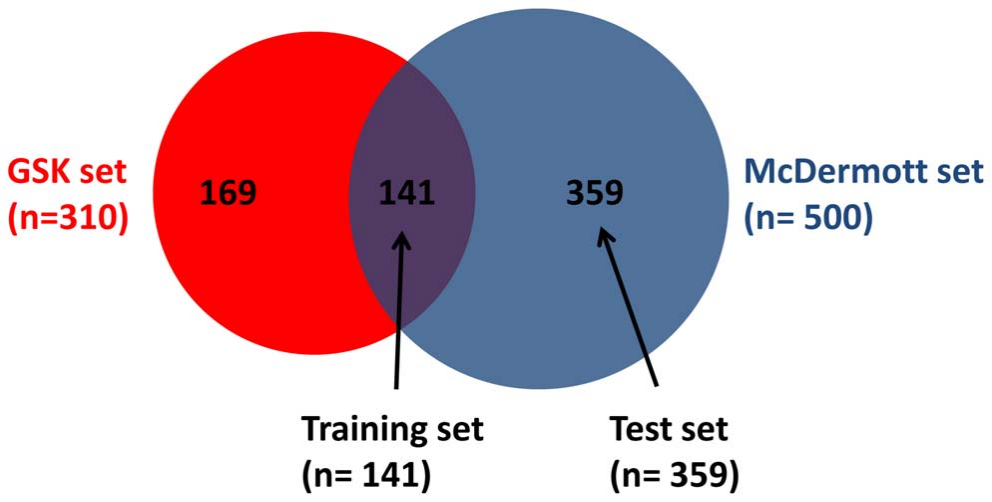
Venn diagram representing the relationships between the GSK and McDermott sets of cell lines analyzed. GSK set is 310 cell lines, genomic info available on 294. McDermott set is 500 cell lines, genomic info available on 366. The 141 cell lines common to the GSK and McDermott were used as a training set, genomic info available on 139.

**Figure 2:**
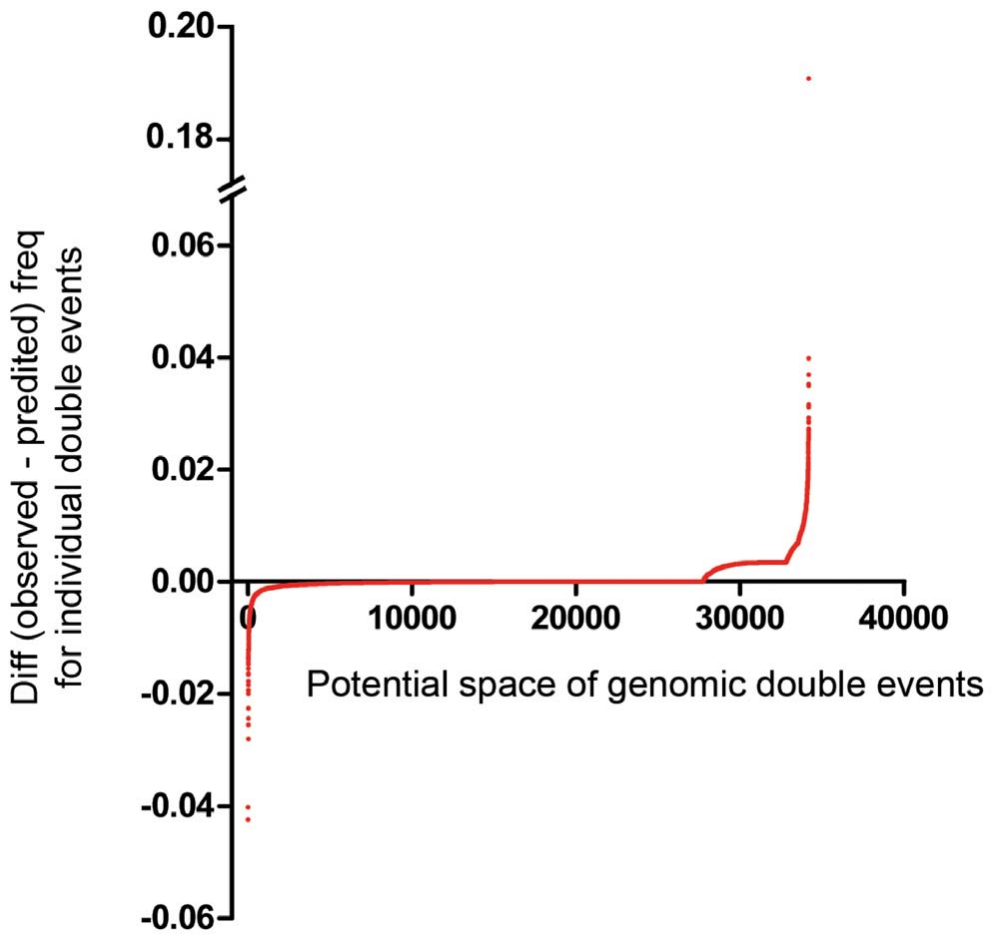
Mutually exclusive events and co-occurring events in 294 cell lines (GSK set). Plot of the difference between the frequencies (*Observed – Predicted)* for all potential double genomic events in the 294 cell lines. Based on 262 distinct genes affected, there were a total of 34,191 potential genomic events involving two genes. Negative differences furthest from zero (left tail) are mutually exclusive events, positive differences furthest from zero (right tail) are co-selected events. The significant events from the left and right tails are found in Table 3 and Table S5 in File S3.

Drug response gIC50 data for a total of 37 targeted compounds tested on 669 cell lines representing the genomic diversity of human cancer types, specifically 23 compounds tested on 310 cell lines (GSK set), and 14 compounds tested on 500 cell lines (McDermott set) were obtained from public databases (Fig. 1) [11]–[13]. Drug response curves were generated and inflection points were mathematically determined based on high order polynomial curve models and defined sensitive vs resistant cell lines for each drug (Fig. S1 in File S1). Cell lines were primarily SNP genotype matched to the Sanger Institute’s Cancer Genome Cell Line database to link drug sensitivity data on cell lines to genomic characterization data available from Sanger [14], including the mutation status from full coding exons sequencing of 64 commonly mutated cancer genes (including copy number), and copy number data from Affymetrix SNP Array 6.0 on 419 genes [14]. A genomic event was defined as either a mutation and/or a copy number aberration in a particular gene. Expected and observed co-event frequency were generated in the sensitive and resistant cell lines and genomic co-features that were in disequilibrium captured through multi-layered statistical and biological significance testing and cross validation thus producing highly significant co-selected and mutually exclusive events including genomic and lineage features in the cell line population and for each drug (Fig. 2). The genomic biomarkers discovered in a 141 cell line training set were independently validated in an independent non-overlapping test set of 359 cell lines screened on 14 of the compounds.

## Methods

**Ethics Statement: N/A.**

### Cell line Growth Inhibition Assays

Data from 23 GSK compounds tested on up to 310 cell lines (range 187–273 cell lines screened per drug, mean = 228 cell lines per drug) were downloaded from resources provided from Greshock and colleagues and Kim and colleagues (GSK set) [11], [13], and data from 14 additional compounds tested on up to 500 cell lines (range 244–500, mean = 460 cell lines) were downloaded from resources provided by McDermott and colleagues (McDermott set) [12] (Fig. 1). Both the GSK and McDermott set of cell lines represent the diverse spectrum of tumor types in human cancer, with 23 and 21 cancer lineages respectively, of epithelial, mesenchymal and hematopoietic origins. For the GSK set, Wooster and colleagues obtained a total of 311 unique cancer cell lines from several vendors (American Type Culture Collection; Developmental Therapeutics Program, National Cancer Institute; German Resource Centre for Biological Material; and European Collection of Animal Cell Cultures), then grown to standard culture media recommended by the vendor; cell lines where authenticated by SNP fingerprinting on Affymetrix 500 K ‘SNP Chip’ as described previously [11]. For the McDermott set, human cancer cell lines were obtained from the American Type Culture Collection (ATCC), the Deutsche Sammlung von Mikroorganismen und Zellkulturen GmbH (DSMZ), the Japanese Collection of Research Bioresources (JHSF), or the European Collection of Cell Cultures (ECACC) and grown according to standard protocols as described previously [12]. Cell line growth inhibition assays were performed as described previously [11], [12]. Briefly, for the GSK set, the midpoint of the growth window (the gIC50) falls halfway between the number of cells at the time of compound addition (T = 0) and the growth of control cells treated with DMSO at 72 hours. The gIC50 value (drug concentration, nmol/L) is a metric for measuring the inhibition of proliferation in cancer cells. Similarly, in the McDermott set, the *cell viability* of each cell line to a given concentration of compound was calculated as the fraction of viable cells to untreated cells present after 72 h of treatment (ratio). We will refer here to gIC50 (GSK set) and cell viability (McDermott set) as Growth Inhibition (GI) values. In both sets, the lower the GI value the more sensitive the cell line to a specific drug.

### Drug Response Curves and Determination of Resistant vs Sensitive Cell Lines

We systematically identified sensitive vs resistant cell lines for each drug. Sensitivity and resistance are not intrinsic properties of the cell lines, but are defined relative to a specific drug. For each drug, we rank ordered and plotted the GI values for the cell line population. The GSK set included 310 cell lines. The McDermott set included 500 cell lines tested on 14 compounds: the 141 cell lines that were common to the McDermott and GSK sets were used as a training set; the remaining non-overlapping 359 cell lines (500–141 = 359) were used as a test set to validate our results on the 14 compound data provided by McDermott (Fig. 1). Based on the determination of the first “inflection point” which corresponds to the area where the graph deviates abruptly into the flat central area of the curve (Fig. S1 in File S1), the cell line population was divided into two groups, the early part of the curve defined the sensitive lines with low GI values (before inflection point), and the flat part of the curve and beyond defined resistant lines with higher GI values (after inflection point). This ensures that cell lines that are defined as sensitive have low GI values and a different sensitivity then the rest of the cell lines tested for that drug. The central flat part of the curve contains cell lines with similar GI values, it corresponds to the peak frequency area on a normal distribution curve when frequency is plotted against GI values. This approach also ensures the effective capture of small subsets of outlier cell lines with marked responses due to low frequency drug-sensitizing genotypes. The drug response curve was modeled mathematically as a high order polynomial curve; the first “inflection point” was determined graphically as the first instance of the largest change in the slope (slope is the first derivative) of the drug response curve, i.e. the first instance of the largest absolute value for the second derivative of the curve (first extremum). Inflection points were determined independently for each compound in the GSK set, and in the training and test sets for compounds in the McDermott set, respectively. In the GSK set, the median gIC50 value at the inflection point was 659 nM, with a range of 16 to 3955 nM. In the McDermott set, we used as a cutoff the inflection point or a GI value of 0.78, whichever gave the greatest sensitivity (smaller GI value): for the training set, the median cutoff was 0.69, with a range of 0.51 to 0.78; for the test set, the median cutoff was 0.74, with a range of 0.50 to 0.78.

### SNP-based Fingerprinting used as Robust Address for Cell Line Cross Referencing

In order to perform genotype-response correlations, we linked genomic information (mutations and copy number aberrations) on cell lines from a separate data set to their response profiles (GI). To avoid potential problems with naming of cell lines and contamination, we used the unique SNP fingerprint of each cell line (GSK set) for cross referencing and matching cell lines across databases. Genotype analysis of the GSK 310 cell line set was performed by SNP fingerprinting on Affymetrix 500 K ‘SNP Chip’ as described previously [11]. Briefly, the SNP fingerprints of the cell lines were compared to each other and to the SNP fingerprint generated by the Wellcome Trust Sanger Institute’s Cancer Genome Cell Line Project as described previously, with cell lines having >80% identity considered a genetic match. There were 283/310 genetically distinct cell lines in the GSK set. Cell viability assays for 25 out of the 37 compounds were restricted to the genetically distinct cell lines. A total of 256/310 cell lines were found to have a genotype match in the Sanger database. Of the genotype matches, 233/256 also matched by name, and for those that did not match by name (n = 23), the genotype association between the names had been previously recorded (http://www.sanger.ac.uk/genetics/CGP/Genotyping/synlinestable.shtml). For the remaining 54 cell lines (310–256), 38 were matched by name to the Sanger database, and 16 remained unmatched (Table S1 in File S3). Cell line names were manually reviewed. Further steps were taken to ensure consistency between cell line names and no duplication in instances were syntax or punctuation differences occurred between names or aliases. For the McDermott set counting 500 cell lines, the 141 cell lines that were common to the McDermott and GSK sets as matched by name were used as a training set, and the remaining non-overlapping 359 cell lines were used as a test set. Cell lines in the test set where a genotype association to a cell line in the training set had been previously recorded were removed from the test set; Additionally, cell lines within the test set where a genotype association had been previously recorded (internal duplicates) were removed with one representative of the cell line remaining. Thus 348 distinct cell lines remained in the test set. A total of 216/348 cell lines were matched to the Sanger database, and 132 remained unmatched (Table S2 in File S3). The genomic characteristics of the corresponding cell lines in the Sanger Cancer Genome Project were downloaded (http://www.sanger.ac.uk/genetics/CGP/CellLines/) for the GSK and McDermott sets respectively; a curated database of the genomic events in each cell line was compiled (Table S3 in File S3 for the GSK set and training set, n = 310–16 = 294; Table S4 in File S3 for the test set, n = 216) based on sequencing data to base-pair resolution of the full coding exons of 64 commonly mutated cancer genes, and genome-wide analysis of copy number gain and loss using Affymetrix SNP 6.0 microarrays and the PICNIC algorithm to predict copy number segments on 419 genes (including the 64 genes above), downloaded from Sanger Cancer Genome Project, Catalogue Of Somatic Mutations In Cancer (COSMIC v51 release) [14]. A genomic event was defined as either a mutation (coding sequence variant), and/or a copy number aberration [a homozygous deletion (total copy number = 0) or an amplification (total copy number > = 8)] in a particular gene. The terms genomic event and mutation are used interchangeably in the text to represent an aberration at a specific genomic site.

### Identifying Genomic Co-events of Relevance

If genomic events (i.e. mutations, copy number aberrations) are randomly distributed in the population of cell lines, and two events co-occur in a cell line independently of each other and due to chance factors alone, and their co-occurrence does not put the cell at a selective advantage or disadvantage, then the predicted co-mutation rate in the population is the following:
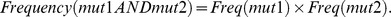



Observed co-mutation rates were compared to predicted co-mutation rates; Co-mutations that occur *more than OR less than* predicted by chance were determined at a significance level of P< = 0.05 by Pearson Chi-square test. These deviations from randomness are likely due to selective pressures. Specifically, to identify co-events that were associated with drug response, we compared predicted to observed frequencies for each drug in sensitive and resistant subpopulations respectively (Chi-square test). When relevant co-mutations were determined in sensitive and resistant subpopulations, in addition to the Chi-square test for statistical significance, a ratio of observed frequencies in sensitive vs resistant lines (S/R) was calculated with a greater than 1.5 fold change [0.667–1.5] being used as a second selection criterion. These approaches were applied to the training and test sets respectively.

### Data Analysis and Clustering

Cluster software was used to adjust GI data prior to hierarchical clustering. For each of the 37 compounds, GI values were first median centered then normalized; this produces a scaled growth inhibition score that is a potency-independent means of comparing response profiles across compounds. The data was then hierarchically clustered using Pearson’s correlation as a metric based on the average distance between nodes. Treeview was used for visualization of the resulting clusters. The Cluster and Treeview software are available from the Eisen laboratory (http://rana.lbl.gov/EisenSoftware.htm) [15].

### Statistical Analysis

Pearson’s Chi-square test (for co-events) and Fisher’s exact test (for single events) were used to assign two-sided P values at 95% confidence interval to describe the correlations between gene mutations/copy number aberrations and drug sensitivity. For the determination of statistically significant single genomic events, with only a few tests run, this obviated the need to correct for multiple comparisons. When the space of two co-events was determined in the 310 cell lines (Table S5 in File S3 and Fig. 2), a Benjamini-Hochberg multiple testing correction threshold with false discovery rate (FDR) of 5% was applied; more than 92% of results remained significant after the correction. Even if for theoretical reasons we apply the most stringent multiple testing correction, the highly conservative Bonferroni correction, to the total unfiltered space of all observed 6871 double co-events, with 6871 tests run, >65% of results remained significant after the correction was applied. More importantly, our biomarker predictions for single and co-events were independently validated in an independent non-overlapping test set of 359 cell lines tested on 14 compounds comprising the McDermott set.

## Results

### Unsupervised Clustering of Drug Response in Cell Lines Recapitulates Pathway Specific Drug Targets and Drivers

**Figure 3:**
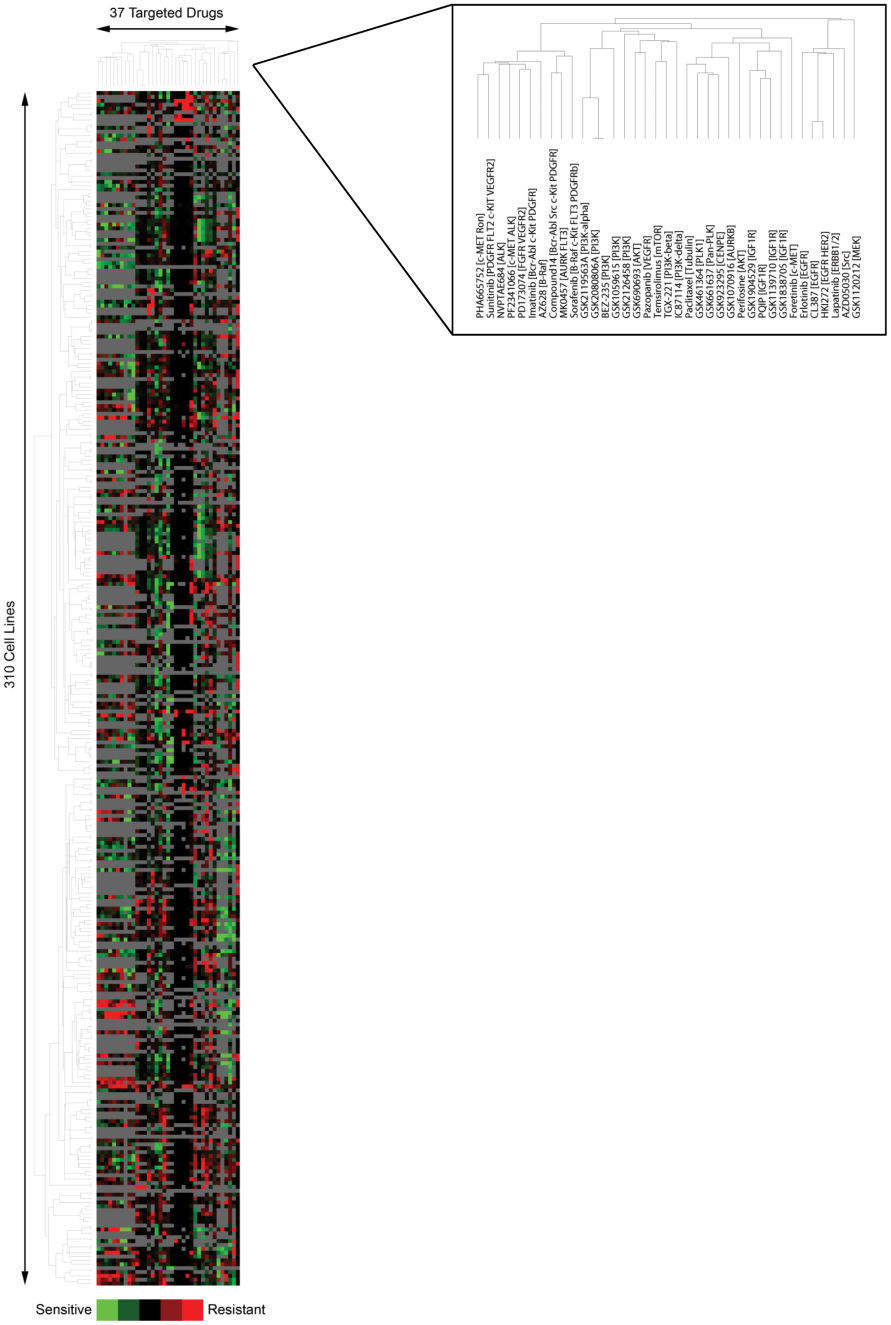
Unsupervised hierarchical clustering of the sensitivity (GI values) of 310 cell lines to 37 targeted drugs. Each row represents a separate cell line, and each column represents a separate compound tested. Increasing sensitivity of a cell line is indicated by the increasing intensity of the green signal, and increasing resistance of a cell line is indicated by the increasing intensity of the red signal; black squares denote sensitivity close to the median across cell lines. Cell lines not screened with a particular compound are indicated in gray. The data from Fig. 3 is split into two less complex figures (Fig.S3 and S4 in File S1) to decrease noise and provide a better display of functional relationships and activated oncogenic pathway subclusters.

We first determined if unsupervised clustering of the sensitivity of 310 human cancer cell lines to 37 targeted drugs could recapitulate known drug mechanisms of action and also the molecular basis of response in highly characterized cell lines. This is visualized in Fig. 3 for a global view of cell line-drug response (and Fig. S2 in File S1). Figs. S3,S4 in File S1 visualize the clustering images for the GSK (23 drugs) and the McDermott sets (14 drugs) separately; the independent analysis reduces the noise introduced by the analysis of 37 compounds and the merging of two independent data sets with different approaches thus yielding a tighter clustering of functional target classes. Indeed, drugs clustered together on the vertical axis according to their main known molecular target. For example, a set of structurally divergent IGF1R targeted drugs clustered in close proximity in Fig. 3 and Fig. S3 in File S1, with a correlation coefficient of 0.78 for the IGF1R subcluster depicted. Hierarchical clustering also recapitulated drug targets within pathways, thus defining pathway specific interventions that can effectively modulate aberrant oncogenic pathways. This was evident in the tight clustering of drugs that target the PI3K/AKT/mTOR pathway, in Fig. 3 and Fig. S3 in File S1, as GSK2126458 [PI3K], GSK690693 [AKT], Temsirolimus [mTOR], TGX-221 [PI3K-beta], IC87114 [PI3K-delta], GSK2119563A [PI3K-alpha], GSK2080806A [PI3K], BEZ-235 [panPI3K and mTOR], and GSK1059615 [PI3K], clustered together with a correlation coefficient of 0.72 for the PI3K/AKT/mTOR pathway subcluster. In Fig. 3 and Fig. S4 in File S1, EGFR and HER2 targeted drugs, Erlotinib [EGFR], CL387 [EGFR], HKI272 [EGFR, HER2], and Lapatinib [ERBB1/2], clustered together with a correlation coefficient of 0.66 for the EGFR subcluster. Mitotic inhibitors, Paclitaxel [Tubulin], GSK461364 [PLK1], GSK661637 [Pan-PLK], GSK923295 [CENPE], and GSK1070916 [AURKB] also clustered closely as shown in Fig. 3 and Fig. S3 in File S1. In this analysis, the lineage of origin of the cells did not significantly influence the organization of the clusters, suggesting that non-lineage dependent events contribute to the response to different drug classes. The molecular lesions that underlie the response to specific drugs in these subclusters are further explored below.

### Single Genomic Events Segregate Sensitive from Resistant Lines

We wanted to identify the molecular basis for the difference in response to drug intervention. We correlated the underlying molecular differences in the cell lines with differences in drug response. To identify which molecular lesions were associated with sensitivity or resistance to a specific drug, the frequency of a genomic event was compared in sensitive vs resistant lines and a ratio of (frequency in sensitive/frequency in resistant), S/R, was computed for any gene aberration present in more than 12% of the sensitive or resistant lines. Any gene frequency that was altered at more than 1.5 fold in sensitive vs resistant lines was considered to be associated with sensitivity to the drug (S/R>1.5) or resistance to the drug (S/R<0.67), respectively. This identified genomic events associated with sensitivity or resistance to specific drug intervention; the statistically significant events are found in Table S6 in File S3.

As an example, lines that were sensitive to the MEK inhibitor GSK1120212 were 2.93 times, 2.35, 1.92, and 1.67 times more likely to harbor a BRAF, KRAS, APC mutation or CDKN2A(p14) mutation, respectively (P = 0.0009, P = 0.0021, P = 0.0243, P = 0.0105), whereas resistant lines were 2.57 more likely to harbor an event in RB1 (P = 0.0024) (Table 1). BRAF and RAS lesions are known to increase MEK activity in tumors [3], [16], therefore interventions that inhibit MEK activity will reduce/normalize downstream signaling through the constitutively activated MEK-ERK kinase cascade and thus these links are expected. However, the association of sensitivity with APC or CDKN2A mutations were not expected and suggest additional biomarkers of response to MEK inhibition.

**Table 1:** Genomic co-events significantly associated with drug response to the MEK inhibitor GSK1120212.

GSK1120212 [MEK]			
Number resistant lines (R)	Number sensitive lines (S)	Total number of lines tested	
107	154	261	
Genomic single events	freq S/freq R	P-value	
RB1	0.389090909	0.002362564	
CDKN2A	1.613741098	0.004576813	
CDKN2A(p14)	1.667532468	0.010530868	
APC	1.924075924	0.024378994	
KRAS	2.351648352	0.002125601	
BRAF	2.933621934	0.000905346	
**Genomic double events**	**freq S/freq R**	**P-value**	**Total number of cell lines with coevent**
MYC::RB1	0	0.010946696	5
ERBB2::PIK3CA	0.138961039	0.043872305	6
EZH2::TP53	0.138961039	0.043872305	6
PIK3CA::PTEN	0.19851577	0.034647786	9
PIK3CA::RB1	0.19851577	0.034647786	9
CDKN2A::PIK3CA	0.260551948	0.05	11
PTEN::RB1	0.308802309	0.043359802	13
RB1::TP53	0.347402597	0.00298131	30
MLH1::TP53	0.434253247	0.04150345	13
PIK3CA::TP53	0.477678571	0.046286597	27
BRCA2::TP53	0.602164502	0.036633993	28
BRCA2::NOTCH1	1.910714286	3.09431E-06	15
KRAS::NOTCH1	1.910714286	0.006373283	15
CDKN2A::PTEN	2.084415584	0.028505985	24
APC::TP53	2.084415584	0.044170186	36
CDKN2A(p14)::PTEN	2.084415584	0.039290204	20
KRAS::TP53	2.238816739	0.020512461	38
BRAF::PTEN	2.316017316	0.023009537	13
SMAD4::TP53	2.605519481	0.011029139	19
BRAF::TP53	3.474025974	0.025865883	24
CDKN2A::KRAS	4.863636364	0.00221916	24
APC::KRAS	5.905844156	0.0062189	19
CDKN2A(p14)::KRAS	5.905844156	0.0062189	19
KRAS::MYC	6.253246753	0.05	10
APC::MYC	6.948051948	0.029972557	11
KRAS::STK11	6.948051948	0.029972557	11
APC::SMAD4	7.642857143	0.030933996	12
APC::BRAF	#DIV/0!/infinite	0.001793855	12
CTNNB1::KRAS	#DIV/0!/infinite	0.022667766	8
FLT3::NOTCH1	#DIV/0!/infinite	0.022667766	8
**Genomic triple events**	**freq S/freq R**	**P-value**	**Total number of cell lines with coevent**
CDKN2A(p14)::FLT3::NRAS	0	0.027313601	4
CDKN2A(p14)::FLT3::TRIM33	0	0.027313601	4
CDKN2A(p14)::NRAS::TRIM33	0	0.027313601	4
CDKN2A::FLT3::NRAS	0	0.027313601	4
CDKN2A::FLT3::TRIM33	0	0.027313601	4
CDKN2A::NRAS::TRIM33	0	0.027313601	4
EGFR::PIK3CA::TP53	0	0.027313601	4
FLT3::NRAS::TRIM33	0	0.027313601	4
PIK3CA::PTEN::RB1	0	0.027313601	4
PIK3CA::PTEN::TP53	0.138961039	0.043872305	6
PIK3CA::RB1::TP53	0.19851577	0.034647786	9
APC::KRAS::TP53	4.863636364	0.017740601	16
APC::MYC::TP53	6.253246753	0.05	10
APC::SMAD4::TP53	6.253246753	0.05	10
APC::BRAF::TP53	#DIV/0!/infinite	0.006248542	10

The top 30 significant single, double and triple genomic co-events are represented here. The frequency of the event in sensitive vs resistant lines is represented by a ratio. #DIV/0!/infinite denotes a strong association with sensitivity. Increasing association with sensitivity in cell lines is indicated by an increasing freq S/freq R ratio >1.5, and increasing association with resistance by a decreasing ratio <0.67, as shown. Top co-events where selected when they fulfilled the following 3 criteria: [1] most significant P-values (P< = 0.05), with [2] the maximum number of cell lines harboring the event, and [3] an S/R ratio that was the furthest from unity in both directions.

Lines sensitive to the AKT inhibitor GSK690693 expectedly harbored mutations in the PI3K pathway, including PIK3CA, PTEN, ERBB2, and also FBXW7, TET2, and BRCA2 alterations (P = 0.0140, P = 0.0197, P = 0.0053, P = 0.0273, P = 0.0346, P = 0.0208, respectively) again suggesting unexpected genomic biomarkers of response to AKT inhibitors that could increase the number of patients likely to benefit; there were no single events significantly associated with resistance (Table S6 in File S3).

PIK3CA aberrations conferred resistance to the BRAF inhibitor AZ628 (P = 0.042) an observation that was confirmed in the test set (P = 0.05), probably through bypass activation of the parallel PI3K pathway, recapitulating results from experimental intervention [17], [18]. On the other hand, BRAF, NRAS, as expected [3], [16], and MLTT3 and MET aberrations conferred sensitivity to AZ628 in the training set (P = 0.003, P = 0.036, P = 0.034, P = 0.003); this was confirmed in the test set for BRAF and NRAS (P = 2.4×10^−10^, P = 0.001) (Table 2).

**Table 2:** Top 30 Genomic co-events significantly associated with response to the BRAF inhibitor AZ628 in the training set (n = 141 cell lines) and validation in the test set (n = 216).

AZ628 [B-Raf]							
Training set				Test set			
N resistant cell lines (R)			121				176
N sensitive cell lines (S)			16				39
Total cell lines tested with drug			137				215
Genomic single events	freq S/freq R	P-value		Genomic single events	freq S/freq R	P-value	
							
PIK3CA	0	0.041992019		PIK3CA	0.188034188	0.05	
BRAF	3.78125	0.003481641		BRAF	7.289940828	2.44481E−10	
NRAS	3.78125	0.0364		NRAS	9.025641026	0.001385118	
MLLT3	5.671875	0.034552321		MLLT3	no power	no power	
MET	6.302083333	0.003490831		MET	no power	no power	
Genomic double events	freq S/freq R	P-value	Number of cell lines with coevent	Genomic double events	freq S/freq R	P-value	Number of cell lines with coevent
APC::PIK3CA	0	0.001387442	12	*APC::PIK3CA*	0	*0.080636657*	6
KRAS::NOTCH1	0	0.05	12	KRAS::NOTCH1	1.128205128	1	5
BRCA2::PIK3CA	0	0.000371807	11	BRCA2::PIK3CA	1.128205128	0.154934007	5
KRAS::STK11	0	0.006134563	11	KRAS::STK11	0	0.311025805	4
MLH1::MSH6	0	9.4913E−16	9	MLH1::MSH6	0	1	1
BRCA2::MLH1	0	6.00151E−07	9	BRCA2::MLH1	0	0.315932049	2
NOTCH1::SMARCA4	0	0.000451726	9	NOTCH1::SMARCA4	0	0.00444622	6
BRCA1::NOTCH1	0	1.88458E−05	8	#N/A	#N/A	#N/A	#N/A
BRCA2::MSH6	0	1.88458E−05	8	BRCA2::MSH6	2.256410256	0.315932049	3
FLT3::PIK3CA	0	0.00346435	8	FLT3::PIK3CA	0	0.044887615	3
MSH6::NOTCH1	0	0.00346435	8	MSH6::NOTCH1	0	1	1
MSH6::PIK3CA	0	0.00346435	8	MSH6::PIK3CA	1.504273504	0.044887615	4
PTEN::RB1	0	0.00346435	8	PTEN::RB1	0	3.18247E−06	11
BRAF::PIK3CA	0	0.041961611	8	BRAF::PIK3CA	0	0.244153005	1
ERBB2::NOTCH1	0	0.041961611	8	ERBB2::NOTCH1	0	1	1
KRAS::SMARCA4	0	0.041961611	8	KRAS::SMARCA4	0	0.311025805	4
BRCA2::CTNNB1	0	0.000363689	7	#N/A	#N/A	#N/A	#N/A
BRCA2::SMARCA4	0	0.000363689	7	BRCA2::SMARCA4	0	1	2
SMARCA4::SMO	0	5.14594E−07	6	SMARCA4::SMO	0	0.03288562	5
SMAD4::TP53	2.26875	0.038867107	13	SMAD4::TP53	0.644688645	0.46785945	8
APC::SMAD4	3.025	0.032429818	7	APC::SMAD4	1.504273504	0.476985713	4
BRAF::TP53	3.78125	0.016854353	15	BRAF::TP53	10.15384615	3.77415E−05	13
BRAF::CDKN2A	4.5375	0.05	8	BRAF::CDKN2A	4.512820513	0.002519772	16
IGK::MET	5.041666667	0.044609724	5	IGK::MET	0	0.315932049	2
APC::BRAF	5.671875	0.034552321	7	*APC::BRAF*	9.025641026	*0.085440703*	3
CDKN2A::MLLT3	5.671875	0.034552321	7	CDKN2A::MLLT3	0	0.315932049	2
MET::TP53	7.5625	0.006713521	8	MET::TP53	0.902564103	0.247216084	12
CDKN2A(p14)::MLLT3	7.5625	0.021295749	6	CDKN2A(p14)::MLLT3	0	1	2
MLLT3::TP53	7.5625	0.021295749	6	MLLT3::TP53	0	0.315932049	2
BRAF::MET	11.34375	0.011484389	5	BRAF::MET	4.512820513	0.15135088	4
Genomic triple events	freq S/freq R	P-value	Number of cell lines with coevent	Genomic triple events	freq S/freq R	P-value	Number of cell lines with coevent
APC::BRAF::TP53	5.671875	0.034552321	7	**APC::BRAF::TP53**	#DIV0!/infinite	**0.032210389**	2
CDKN2A::MLLT3::TP53	7.5625	0.021295749	6	CDKN2A::MLLT3::TP53	0	1	2
BRAF::MET::TP53	11.34375	0.011484389	5	*BRAF::MET::TP53*	9.025641026	*0.085440703*	3
CDKN2A(p14)::MLLT3::TP53	11.34375	0.011484389	5	CDKN2A(p14)::MLLT3::TP53	0	1	2
BRAF::SMAD4::TP53	5.041666667	*0.1*	5	BRAF::SMAD4::TP53	#DIV0!/infinite	0.181395349	1
IGK::MET::TP53	5.041666667	*0.1*	5	IGK::MET::TP53	0	1	2
APC::BRAF::SMAD4	7.5625	*0.066988658*	4	#N/A	#N/A	#N/A	#N/A

The frequency of the event in sensitive vs resistant lines is represented by a ratio (freq S/freq R). Events where the ratio is <0.67 are associated with resistance to the drug, and events where the ratio is >1.5 are associated with sensitivity. “#DIV0!/infinite” denotes a strong association with sensitivity. Under Test Set, numbers in **BOLD** indicate events that are significantly associated with S or R: P< = 0.05, AND S/R < = 0.67 or > = 1.5, AND S/R concordant with that of training set. Numbers in *ITALICS* under Test Set indicate the same but P-value [0.06–0.10]. “No power” for single events under Test Set indicates less than 12% occurrence of the single mutation of interest in the sensitive and resistant lines respectively in the test set. “#N/A” indicates the co-event did not occur for the cell lines studied in the test set. 6/30 (20%) double genomic events that were predictive in the training set were also predictive in the test set at P< = 0.05, and 8/30 (27%) were predictive at P< = 0.10.

Although there was a strong association of single genetic aberrations with response to therapeutic agents, this association was not absolute with a number of cell lines containing any specific event being scored as either sensitive or resistant. Thus there must be additional events that cooperate with the mutational status to determine sensitivity and resistance to targeted therapeutics. Other than the associations noted above and aberrations in PTEN and ERBB2 potentially contributing to the clustering of EGFR family inhibitors and aberrations in PTEN and CDKN2A being associated with drugs targeting the PI3K and IGF1R pathways, there were no clear aberrations driving the majority of the drug response clusters (Fig. 3). Again this suggests that additional co-events must contribute to drug sensitivity and resistance. The potential co-events are explored below.

### Molecular Co-occurring Events Reveal Drivers and Co-actionable Targets in Cancer

**Figure 4:**
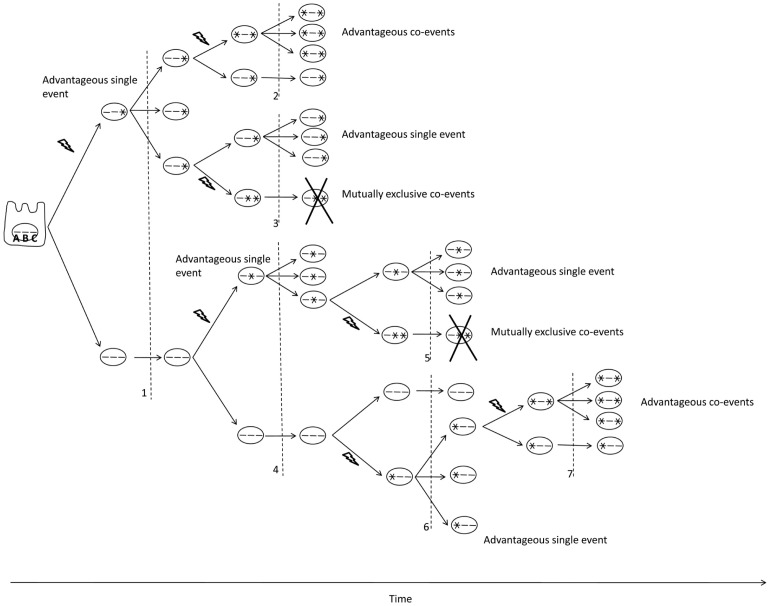
Cancer as a micro-evolutionary process. Illustrated here is a sequential multiple hit model of cancer initiation and progression, underlying co-selection and mutual exclusivity of genetic events in cancer. A, B, C are genes inside a cell’s nucleus. After a mutation occurs (marked by lightning strike), the progeny of a cell are subjected to selective pressures as illustrated in 1, 2, 3, 4, 5, 6 and 7. Mutations that provide a survival and proliferative advantage to the cell will lead to an increase of that cell and its gene pool in the population of cells. The cell with a mutation in gene C has an advantage over the cell with no mutation and it will outnumber the latter. The same applies to the cell with mutation in genes A or B respectively. Subsequently, the cell with sequential mutations in genes A and C has a proliferative advantage over the cells with either mutations A alone or C alone [7 and 2], and it will outnumber them; these cells carry advantageous co-mutations that are co-selected in the population. Conversely, the cell with mutations in B and C, even though each mutation on its own endows a proliferative advantage [4 and 1], is at a selective disadvantage as compared to cells with mutations in B only [5], or C only [3], and therefore will tend to disappear from the cell population; these cells harbor mutually exclusive co-events.

To identify potential cooperating events, we first identified genomic events that co-occurred beyond what is expected if they were independent and the association due to chance factors alone. This defines co-events that were likely under selective pressure during tumor initiation or progression (or during adaptation to culture) and thus have a high probability of being drivers of the cancer phenotype. Molecular lesions whose co-occurrence leads to a proliferative or survival advantage beyond what is observed for separate single occurrences will be co-selected and the frequency of the co-event will increase in the population (Fig. 4). Based on the database of genomic events in cell lines, the space of observed two co-events (e.g. mut1-mut2) was generated for the 294 cell lines for which genomic information was available (GSK set). There were 6871 observed distinct double co-events in the 294 cell lines, and 12958 total occurrences. There were 95415 distinct triple co-events (e.g. mut1-mut2-mut3) in the 294 cell lines, and 110872 total occurrences. We compared predicted to observed co-event frequencies in the 294 cell lines where sufficient numbers of co-events were available to provide statistical analysis (Fig. 2; Table S5 in File S3).

To determine whether co-events predicted response to therapeutic agents, we compared predicted to observed frequencies for each drug in sensitive lines and resistant lines respectively, and also determined which co-events occurred at different frequencies in sensitive vs resistant lines (Table S6 in File S3). For the MEK inhibitor GSK1120212, co-events involving two genes CTNNB1::KRAS, APC::BRAF, APC::SMAD4, KRAS::STK11, APC::MYC, APC::KRAS, BRAF::TP53, BRAF::PTEN were associated with sensitivity, whereas co-events MYC::RB1, ERBB2::PIK3CA, PIK3CA::PTEN, RB1::TP53, MLH1::TP53 were associated with resistance (Table 1) (P< = 0.05). While the co-events involving KRAS and BRAF aberrations would potentially be identified through prior knowledge, the association of sensitivity to the MEK inhibitor with coordinate events in APC::SMAD4 and APC::MYC as well as the set of resistance mutations would not be predicted. Interestingly some aberrations were found as partners in association with both sensitivity and resistance such as MYC, TP53 and PTEN suggesting that markers of sensitivity and resistance may be context dependent. Triple co-events involving three genes APC::BRAF::TP53, APC::SMAD4::TP53, APC::KRAS::TP53 were associated with sensitivity, and CDKN2A::FLT3::NRAS, PIK3CA::PTEN::RB1, EGFR::PIK3CA::TP53 were associated with resistance to the MEK inhibitor (Table 1) (P< = 0.05).

For the AKT inhibitor GSK690693, co-events involving two genes CDH1::ERBB2, FBXW7::PTEN, FLT3::MSH6 and BRCA2::PALB2 were associated with sensitivity, while BRAF::CDKN2A, KRAS::MYC, KRAS::SMO were associated with resistance (Table S6 in File S3). These results for concomitant mutational activation of KRAS and MYC are consistent with evidence that MYC activity downstream of AKT would bypass AKT normalization downstream of activated RAS and resistance would ensue [17]. Triple co-events FBXW7::PTEN::TP53, FLT3::MSH6::TP53, BRCA2::PALB2::PIK3CA were associated with sensitivity to GSK690693.

For the BRAF inhibitor AZ628 (Table 2, Table S7 in File S3) double genomic events NOTCH1::SMARCA4, FLT3::PIK3CA, PTEN::RB1 and SMARCA4::SMO were associated with resistance to the intervention in the training and the test sets (P = 0.004, P = 0.045, P = 3×10^−6^, P = 0.033, test set), while BRAF::TP53 and BRAF::CDKN2A were associated with sensitivity in both sets (P = 4×10^−5^, P = 0.003, test set). These results are consistent with recent demonstration that RAF inhibitors inhibit ERK signaling in cells with mutant BRAF but unexpectedly enhance signaling in cells with wild-type BRAF, and that KRAS activation appears to promote RAF dimerization and resulting MEK activation, compatible with resistance seen here in the context of KRAS::NOTCH1, KRAS::STK11 and KRAS::SMARCA4 (P< = 0.05, training set only) [3].The combination of APC::BRAF::TP53 was associated with sensitivity in both the training and test sets, and BRAF::MET::TP53 showed sensitivity in the training set (P = 0.01) and a trend toward sensitivity in the test set (P = 0.085).

For HKI272, an EGFR/HER2 inhibitor, MLH1::SMO, BRCA2::MSH6, PTEN::RB1 were associated with resistance in both the training and test sets (P = 0.003, P = 0.045, P = 3×10^−6^, test set), while ERBB2::TP53, KDR::STK11 (P = 0.043, P = 0.003, test set) were associated with sensitivity to the intervention (Table S7 in File S3). The occurrence of CDKN2A, ERBB2 and TP53 aberrations together predicted sensitivity in the training and test sets (P = 0.04). PTEN loss and PI3K pathway activation are known to be correlated with resistance to EGFR/HER2 blockade [5], [6]; Interestingly, if the observation that ERBB2 amplification in the context of TP53 inactivation is associated with sensitivity holds with further experimentation, then dual testing for these mutations could help stratify patients who would most likely benefit from this intervention.

This process was repeated for each of the 37 targeted drug interventions, and for the 14 drugs tested on 359 independent cell lines in the test set, and co-events that were significantly associated with sensitivity or resistance were tabulated in Table S6 in File S3. Table S7 in File S3 compares significant biomarkers in the training and test sets: multiple co-events were confirmed to be predictive in the training and test sets. Low numbers of cell lines harboring specific co-events even in the large number of cell lines examined, resulting in low power, might have prevented the identification of significant co-biomarkers with confidence for some of the drugs, in particular the limited number of cell lines observed to be sensitive for many of the drugs tested. In addition, even though cell lines were primarily SNP genotype matched to link drug activity to genomic data across databases, matching inevitably introduces error; ideally the same experimental batch of cell lines should be split into a set tested on drugs and a set that receives genomic characterization.

Within the drug classes, there are combinations that occur recurrently. This suggests that combinations of mutations have a greater ability to predict response and sensitivity to classes of drugs than do single mutations. For example, for the EGFR family inhibitors, combinations of BRCA1, BRCA2, NOTCH1, MLH1 and CDKN2A are strong predictors. For the IGF1R family of inhibitors, combinations with PTEN, BRCA2 CDH1, are indicators of sensitivity and combinations of KRAS, BRAF, TP53, and PIK3CA are indicators of resistance. For the PI3K family of inhibitors aberrations in NRAS, BRCA1/2 and PTEN mediate sensitivity while combinations with KRAS, MYC, and different combinations with PTEN mediate resistance. Intriguingly, aberrations that can alone be indicators of sensitivity appear to mediate resistance when combined with other aberrations, e.g. the effect of combinations with PTEN in PI3K pathway inhibitors.

The observation that there are fewer cooperating events involving three genes is likely due to the diminished power to observe these events even in the large number of cell lines studied. Nevertheless, a number of coordinate events proved to have markedly stronger predictive value than single events alone suggesting that interactions between multiple events are critical determinants of response to targeted therapeutics.

### Mutually Exclusive Events Reveal Actionable Targets in Cancer

Multiple aberrations in cells can occur together at greater, expected, or lower than expected frequencies based on the frequency of single events. The presence of disequilibrium from expected frequencies provides evidence for selection during tumor initiation or progression with for example events that occur at lower than expected frequencies likely representing liabilities to the cell (Fig. 4). By examining a large number of cell lines, genomic events that occur individually above the background mutation rate but do not co-occur in any one tumor can be identified; those are mutually exclusive events and are subject to selective pressures. We have identified in this set of cell lines all genes that occur together in a pair less than predicted by chance; We defined mutually exclusive events as those that do not occur together (observed frequency = 0), and partially exclusive events as those that occur together at lower than predicted frequency. Mutually exclusive events can define cancer drivers with high probability and likely targets for intervention (see Discussion). We computed the potential space of all two co-events in 294 cell lines (GSK set) and then calculated the difference between the frequencies *Observed – Predicted* for each co-event. Based on 262 distinct genes affected, there were a total of 34,191 potential two co-events [combinations of 2 in 262 = 262!/(2!(262-2)!)], and 6871 observed two co-events. Negative differences furthest from zero are mutually exclusive events where the observed frequency is zero; the rest are partially exclusive events (Fig. 2, Table 3, Table S5 in File S3). For example, CDKN2A and IGH are mutually exclusive events (P = 0.044), as well as MYC and TET2 (P = 0.044) in the 294 cell lines examined. CDKN2A and EZH2, CTNNB1 and MYC, IDH1 and PTEN, and NRAS and TET2 showed a trend toward mutual exclusivity (P = 0.081 for all). On the other hand, APC::CDKN2A, CDKN2A::RB1, KRAS::RB1, and CDKN2A(p14)::SMARCA4 all occurred together at frequencies lower than predicted by chance (P = 0.005, P = 0.046, P = 0.010, P = 0.05, respectively), whereas CDKN2A::PIK3CA, KRAS::PTEN (P = 0.089, P = 0.077, respectively) showed a trend toward partial exclusivity. In the independent 348 cell line test set, CDKN2A and RB1 were also found to be partially exclusive, as well as CDKN2A(p14) and RB1 (Table S8 in File S3). Mutations in the enzyme isocitrate dehydrogenase 1 (IDH1), common in a major subset of primary human brain cancers were recently shown to produce the oncometabolite 2-hydroxyglutarate with a role in the formation and the malignant progression of glioma; PTEN is also commonly lost in glioblastoma [19]–[21]; Our results suggest that they occur together less than would be expected by chance. Furthermore, all the events (except for IGH) that make up the mutually exclusive event pairs in Table 3 were significantly associated with sensitivity or resistance to the specific drugs as indicated in blue for the single genomic events in Table S6 in File S3, further supporting an important driver role in neoplasia. Thus, as discussed further below, the genes that make up these pairs probably play, each on its own, an important role in cancer and could be used as individual or co-targets for intervention (see Discussion). In Table S6 in File S3, for each drug, by design, most of the co-selected events found in sensitive lines were reciprocally partially exclusive in the resistant lines and vice versa (Table S9 in File S3); this allowed for a stronger mechanistic basis for the identification of co-events and drivers in cancer. When we then looked separately at partially exclusive events for specific drugs in the total population of cell lines studied (GSK set, n = 294), we found that partially exclusive events were rare occurrences as compared to co-selected events, as was expected from Table S5 in File S3. Specifically, for the MEKi GSK1120212, only APC::CDKN2A (P = 0.004) were partially exclusive and significantly associated with sensitivity to this drug (Table S10 in File S3). For MK0457, APC::CDKN2A (P = 0.002) were associated with resistance, as well as KRAS::PTEN (P = 0.01), while KRAS::RB1 was associated with sensitivity (P = 0.04). A co-event that is associated with one phenotypic response to a drug (sensitivity), can be associated with the opposite response to another drug (resistance). As compared to our work, limited efforts by other groups have used different definitions, goals and approaches to define mutually exclusive events that were based on prior biological knowledge as opposed to our unbiased data-driven approach, were cancer specific, limited in scale and not designed to demonstrate mutually exclusive events driving tumorigenesis across cancer types or pathways, and importantly have not linked mutually exclusive genetic aberrations to drug sensitivity [22].

**Table 3:** Mutually exclusive events in 294 cell lines (GSK set).

Potential space of double coevents	Observed freq coevents	Predicted freq coevents	Difference (freq obs - freq predicted)	Cell lines with coevent (observed)	Cell lines with no coevent (observed)	Cell lines with coevent (predicted)	Cell lines with no coevent (predicted)	P-value Chi-square test
APC::CDKN2A	0.027210884	0.069623768	−0.042412884	8	286	20	274	0.005444485
APC::CDKN2A(p14)	0.017006803	0.057233097	−0.040226295	5	289	17	277	0.002713951
CDKN2A::RB1	0.030612245	0.058702393	−0.028090148	9	285	17	277	0.045615078
KRAS::RB1	0.006802721	0.032336064	−0.025533343	2	292	10	284	0.010053727
CDKN2A(p14)::RB1	0.023809524	0.048255357	−0.024445833	7	287	14	280	0.05
CDKN2A::PIK3CA	0.037414966	0.060067564	−0.022652598	11	283	18	276	*0.088592844*
CDKN2A(p14)::SMARCA4	0.013605442	0.033666528	−0.020061086	4	290	10	284	0.05
KRAS::PTEN	0.020408163	0.039856078	−0.019447915	6	288	12	282	*0.076974189*
CDKN2A::MSH6	0.017006803	0.03549447	−0.018487667	5	289	10	284	*0.1*
CDKN2A(p14)::MSH6	0.013605442	0.029177657	−0.015572215	4	290	9	285	*0.090497767*
KRAS::NRAS	0.003401361	0.017296034	−0.013894674	1	293	5	289	*0.07119017*
CDKN2A::DICER1	0.006802721	0.019112407	−0.012309686	2	292	6	288	*0.098960185*
**CDKN2A::IGH**	0	0.012286547	−0.012286547	0	294	4	290	0.044036228
**MYC::TET2**	0	0.012217132	−0.012217132	0	294	4	290	0.044036228
**CDKN2A(p14)::IGH**	0	0.010099958	−0.010099958	0	294	3	291	*0.081691311*
**CDKN2A::EZH2**	0	0.009556203	−0.009556203	0	294	3	291	*0.081691311*
**CTNNB1::MYC**	0	0.009162849	−0.009162849	0	294	3	291	*0.081691311*
**IDH1::PTEN**	0	0.008584386	−0.008584386	0	294	3	291	*0.081691311*
**NRAS::TET2**	0	0.008514971	−0.008514971	0	294	3	291	*0.081691311*

In the potential space of double genomic events totaling 34191 co-events, we identified events that co-occurred less than expected by chance. These correspond to the extreme left tail of the distribution in Fig. 2. Events that co-occur less than expected by chance are mutually exclusive (observed freq = 0; **BOLD**), or partially exclusive (observed freq>0). Events with the most significant P-values AND maximal differences are reported here. *Italics* indicate P-values [0.06–0.10].

### Lineage Combined with Mutation can Predict Response to Drugs

As indicated above, although there is an association between single mutations and response to therapeutic agents, this association is weaker than expected. To determine whether additional co-events could increase the predictive value, we determined whether a combination of mutation and lineage would be a stronger predictor than single mutations.

We first determined if mutation-lineage pairs (mutation is used here for any genomic event) in cell lines were associated with a specific drug response phenotype. For the 294 cell lines (GSK set) for which genomic information was available, we computed the occurrence of each mutation-lineage pair; there were 2132 such pairs (e.g. BRAF::Melanoma). We tabulated all the lineages that co-occurred with each mutation (e.g. BRAF::Melanoma, BRAF::Colon, etc), and for each drug how many of each pair occurred in sensitive vs resistant lines (e.g. BRAF::Melanoma in sensitive lines = 14; BRAF::Melanoma in resistant lines = 0), and compared that, respectively, with the chance random distribution between sensitive and resistant, at P< = 0.05.

As an example, we examined the response of tumors harboring the same mutation across lineages to the MEK inhibitor GSK1120212. The single aberrations that were significantly associated with response above (Table 1) were tested one at a time and their interaction with each lineage in determining sensitivity evaluated (each mutation1-lineageX pair was tested individually and also together after lineages that had reached significance were subtracted from the sample. This provides an internal test set for cross validation as the remaining cell lines were not used in identifying the mutation1-lineageX pair that interacts significantly with response) (process flowchart in Fig. S5 in File S1). Thus, for the MEK inhibitor, BRAF mutation was associated with sensitivity when it occurred in the context of colon cancer and melanoma, while there were no association noted when it occurred in the context of other organ sites (Table S11 in File S3). As another example, events in APC were found to be associated with sensitivity to the MEK inhibitor GSK1120212 in colon cancer cell lines, and there were no associations in the context of lines from other organ sites. Strikingly, PIK3CA aberrations were associated with sensitivity to the MEK inhibitor when it occurred in colon cancer lines, and resistance when they occurred in breast cancer lines; there were no associations noted in the context of other lineages. Finally RB1 was associated with resistance to the MEK inhibitor when it occurred in cell lines from lung cancer, but there were no associations with response phenotype noted when it occurred in the context of other lineages. Thus, we found that single mutations appeared to be sensitizing only in the context of specific lineages, while there were no associations or even inverse associations with response phenotype noted in the presence of the same mutation in other lineages.

These observations were reproduced when tested in other drugs. For the AKT inhibitor GSK690693, PIK3CA aberrations predicted resistance only in colon cancer cell lines, and PTEN mutations predicted resistance in CNS and melanoma cell lines but there were no associations when they occurred in other lineages (Table S12 in File S3). For the BRAF inhibitor AZ628, PIK3CA mutation predicted resistance when it occurred in the context of a breast cancer or colon cancer cell line (training set). Similarly, in the test set, PIK3CA mutation predicted resistance when occurring in a breast cancer cell line. For the EGFR/HER2 inhibitor HKI272, APC mutation was associated with resistance when occurring in colon cell lines, PTEN appeared to confer resistance in the context of breast and CNS lines; these observations were confirmed in the test set (Tables S13–S18 in File S3).

While the association between lineage and mutation proved a stronger predictor than lineage or mutation alone, in many cases the associations remained weak and in some cases such as BRAF and sensitivity to the MEK inhibitor in colon cancer have not been validated in clinical studies [23]. We thus sought additional explanations for the sensitivity patterns to different drugs.

### Co-mutations Explain the Effect of Lineage in Predicting Response to Therapy

Lineage could be a predictor through a selection of particular combinations of mutations that occur in a specific tumor lineage (Fig. S6 in File S1, and Fig. S7 in File S2). Thus we determined whether genomic co-events superseded the predictive value of lineage. We determined whether comutations or lineage were superior predictors of response to a drug (flow diagram Fig. S5 in File S1). This is illustrated for the MEK inhibitor in Table S19 in File S3. For each of the 30 significant co-mutations (two genes) from Table 1 (or Table S6 in File S3), we examined the interaction of the co-mutation and each lineage in determining the response (S vs R) to the MEK inhibitor. To test our hypothesis and because of the low power for occurrence of identical co-mutations in a specific lineage due to limited numbers of cell lines per lineage, we used cross validation with an internal test set approach that was not contaminated by the selection of significant markers from Table S19 in File S3; we excluded cell lines from tumor lineages that interacted with any of the co-mutations in Table 1 and only considered cell lines that were not used to identify the significant comutation-lineage interactions (Table S19 in File S3). We examined each co-mutation pair in the context of all observed lineages, and determined if the mutation pairs were significantly associated with the same drug response phenotype regardless of lineage. For each co-mutation pair, the individual lineages that were interacting significantly were subtracted from the total occurrence for that co-mutation and we assessed if the association with drug response remained significant for the other lineages. We identified a series of co-mutations and lineages that were predictions. Out of the 30 significant co-events examined for the MEK inhibitor, 27 had sufficient power in the remaining cell lines to determine the effects of co-mutations. 9/27 (33%) co-events were significantly associated with drug susceptibility regardless of lineage at P< = 0.05; for the other co-events there were no associations with drug response. 17/27 (63%) co-events showed an association with drug susceptibility across lineages at P< = 0.10. Of the 4 co-events that involved RB1, only 1 reached significance at P< = 0.05; of the 9 co-events that involved p53, only 2 reached significance at P< = 0.05. If co-events involving TP53 or RB1 are removed, 6/14 (43%) co-events are associated with drug response at P< = 0.05, and 14/14 (100%) at P< = 0.10.

For AZ628, presence of APC::PIK3CA, BRAF::PIK3CA, BRCA1::NOTCH1, BRCA2::MLH1, BRCA2::PIK3CA, BRCA2::SMARCA4, ERBB2::NOTCH1, FLT3::PIK3CA, NOTCH1::SMARCA4, PTEN::RB1, SMAD4::TP53, SMARCA4::SMO, or KRAS::NOTCH1 were associated with resistance to this BRAF inhibitor independent of lineage at P<0.05 (13/30 = 43%) (training set, Table S20 in File S3). In addition, BRCA2::CTNNB1 (P = 0.083), KRAS::STK11 (P = 0.083), MLH1::MSH6 (P = 0.083), MSH6::NOTCH1 (P = 0.083) were associated with resistance independent of lineage at P<0.10 (total 17/30 = 57%). When the same significant co-mutations from Table S6 in File S3 were analyzed for interaction with lineage in the 348 cell line test set, APC::PIK3CA, NOTCH1::SMARCA4, PTEN::RB1, SMAD4::TP53 were also associated with resistance independent of lineage as in the training set (Table S21 in File S3). FLT3::PIK3CA showed a trend for resistance at P = 0.083. KRAS::STK11 was significantly associated with resistance in the only lineage in which it occurred (lung) which precluded analysis across lineages.

For the HKI272, APC::TP53, BRCA1::NOTCH1, ERBB2::NOTCH1, MLH1::SMO, PTEN::RB1, SMAD4::TP53 were associated with resistance to this EGFR/HER2 inhibitor independent of lineage at P<0.05. In addition, MSH6::NOTCH1 (P = 0.083) showed a trend to resistance regardless of lineage (training set, Table S22 in File S3). This was confirmed in the test set for MLH1::SMO, PTEN::RB1 at P<0.05, and for APC::TP53 at P< = 0.10 (Table S23 in File S3). Furthermore, significant coevents APC::SMAD4, BRCA2::CDKN2A, BRCA2::TP53, MSH2::TP53, RB1::TP53 predicted resistance to HKI272 across lineage at P<0.05, an association that was limited to the test set. The interactions of co-mutations and lineage were tested in additional drugs including the AKT inhibitor GSK690693 and MK0457, further supporting the above findings that co-mutations predict sensitivity across lineages (Tables S24–S26 in File S3).

Therefore, the interaction of two genomic events can determine the response to a targeted intervention independent of lineage. This suggests that an important effect of lineage is potentially in determining the sets of co-mutations that occur, and that the co-mutations are the dominant predictor. Low power due to the limited number of cell lines with a specific co-mutation in a specific lineage could have limited the identification of further significant co-mutations predictions in the training and test sets.

## Discussion

Translating the cancer genome into highly efficacious targets to improve patient outcomes is a major emerging challenge. The last ten years in cancer research have delivered many breakthroughs, among them, first, the demonstration that oncogene-addicted tumors can be specifically targeted with single agents to successfully halt and revert cellular proliferation, at least transiently [1], [2], [4], [24]; and second, the comprehensive characterization of tumors at the molecular level through efforts spearheaded by the TCGA and ICGC. The emerging challenge is how to leverage the accumulated molecular data in terms of interpretation and validation efforts that will deliver robust drivers and targets for effective therapeutic intervention. To begin to respond to this challenge we have linked large scale genomic information and response data from high throughput drug screens in human preclinical model systems to identify a set of potential predictors of response to targeted therapeutics. Specifically, we demonstrate that a pipeline composed of a scalable in silico bioinformatic platform coupled to high throughput cancer cell line models for functional genomic discovery and validation could act as an interface between molecular characterization efforts on one end and the clinic/clinical trials on the other. By applying this pipeline to 669 highly characterized cancer cell lines, we demonstrate that combinations of mutations are potentially powerful biomarkers of response for targeted therapeutics. Thus, assessing combinations of biomarkers could identify patients most likely to benefit from targeted therapy. Further, as the molecular events interact to predict response to individual drugs, they may represent targets for simultaneous combinatorial intervention. Thus, when applied in concert with the imminent widespread democratization of molecular typing of patient tumors in the clinical setting, this platform holds the promise to improve patient outcomes by delivering critical mechanistic information that could form the basis for personalized biomarkers and combinatorial therapy in molecularly matched patient subsets. We chose genomic DNA as a biomarker because DNA is relatively stable as compared to other information content of the cell, and because for diagnostic purposes sequencing technologies have reached the maturity and affordability to be used in the clinic, and can be readily applied to fresh or archived clinical specimens from tissue or blood (http://www.cancergenome.nih.gov/) [25]. Two recent reports in Nature examine biomarkers of response in 639 cell lines and 130 drugs (Sanger Institute group), and 479 cell lines and 24 drugs (The Broad Institute group), respectively [26], [27]. Using these overlapping sets of cell lines, also largely overlapping with our set, they link individual genomic or gene expression features in cell lines with response to drugs, or individual genomic features with individual gene expression or tissue type features to predict response. This work is different from and complements our effort as it does not seek to systematically identify combinations of genomic biomarkers as predictors of response, nor does it address the question of the identification of combinatorial drivers and targets from large scale genomic datasets, which we have both addressed here.

Single targeted interventions have had limited success in cancer [1]–[6], [28]. Initial successes with single agent kinase inhibitors against oncogene-addicted tumors have been stunted by the emergence of resistance and the realization that oncogene-addicted tumors account only for a small fraction of all neoplasms. Rare tumors are addicted to single activated oncogenes, recently exemplified by bcr-abl in CML, ALK in 4% of NSCLC, and BRAF in certain melanomas [1]–[4], [24], [29]. While these molecular lesions offer a highly druggable target, in most cases where drugs and biomarkers have been linked, the tumors often quickly develop resistance with resultant therapeutic failure. There are several compelling reasons why combinatorial drug approaches will be needed to overcome resistance in cancer: (1) tumor heterogeneity in the individual, (2) more than one cancer driver at play, and (3) homeostatic feedback loops in the cancer cell. Biological systems are robust to single perturbation due to functional redundancy and multiple homeostatic feedback loops that maintain a stable intracellular environment in the face of perturbation [3], [5], [25]. Targeting one node in a pathway is frequently not sufficient to shutdown the pathway. Further, multiple molecular aberrations can cooperate to ensure dysregulation of an oncogenic pathway [6], [30]. Recent clinical studies have demonstrated that MET amplification predicts that patients with lung cancer and activating EGFR mutations will fail to respond to erlotinib or gefitinib, and KRAS mutation predicts that patients with colon cancer will fail to respond to cetuximab [31], [32]. In patients with glioblastoma multiforme, a mutation affecting the EGFR receptor predicts sensitivity to EGFR inhibitors only when the tumor suppressor PTEN is also intact [19]. These studies illustrate the complex role of signaling events and critical co-mutations in predicting response to therapy. There is a need to move away from single gene-based therapeutics with low predictive value and high emergence of resistance, to combinatorial co-targeting with high predictive value and low emergence of resistance because of a mechanistically guided targeting of multiple nodes along activated pathways. We have identified systematically and on a large scale a series of co-targets that may be susceptible to synthetic lethal approaches. Co-occurring and mutually exclusive molecular events offer a framework for the selection of rational drug combinations in cancer that can increase the predictive value for response and minimize the emergence of resistance. These approaches define highly efficacious targets with low potential for resistance intervention.

One of the major emerging challenges post data production from massively parallel sequencing is the need to differentiate “driver” from “passenger” mutations. Genetic aberrations that are conserved horizontally across individuals (i.e. hot spot mutations) demonstrate selection for a change in function and identify genomic aberrations that play an important role in tumorigenesis providing one mechanism for identifying driver mutations. Cancer is a microevolutionary process (Fig. 4). We used evolution as a filter to parse driving aberrations from noise. The initial occurrence of a mutation is a more or less random event. This random event is followed by a selection process that shapes the combinations of mutations that accumulate in the population of cells and which mutations disappear. Genetic aberrations that put a cancer cell at a proliferative and survival advantage in a specific microenvironment (e.g. breast cancer metastasis in the liver; patient receiving targeted therapy) will tend to accumulate in the cell population. Using this concept, we have identified event pairs that are in disequilibrium and likely cooperate to increase the number of cells in the population. Both members of the event pairs are likely driver mutations and therefore either they or their downstream effector (for example in the case of an inactivated tumor suppressor) constitute potential targets for combinatorial intervention. Frequency plots of genetic alterations at the output stream of sequencing efforts in cancer reveal a mountain of a few but frequent aberrations (e.g. EGFR, CDKN2A, TP53 and PTEN in glioblastoma, TP53, PI3K and HER2 in breast cancer, BRAF and NRAS in melanoma) and a long tail of many low frequency aberrations (http://www.cancergenome.nih.gov). Our data suggests that combined targeting of specific co-aberrations *in the mountain and in the tail* will be needed to maximize response and minimize the emergence of resistance.

As opposed to the Knudson double hit theory where two consecutive hits are required to initiate cancer [33], in the case of mutually exclusive mutations, the mutation combination (A AND B) appears to engender a survival and proliferative disadvantage as compared to a cancer cell that only carries one of the two hits, and these cells would tend to disappear from the population, leading to the observed mutual exclusivity in the gene pool (Fig. 4). Mutations A and B respectively occur in separate cancers above the background mutation rate; If the functional state resulting from mutations A AND B occurring together perturbs the intact function of mutation A alone (e.g. oncogenic activation) OR mutation B alone and is disadvantageous and maybe lethal to the cancer cell, this implies that the intact function of mutation A alone OR B alone is needed for cancer proliferation and survival in cells that harbor these respective mutations; so A and B are driver mutations by definition and possibly synthetic lethal targets for intervention.

It is expected that the emerging understanding of the molecular mechanisms of cancer will transform the clinical trial landscape from a trial and error system to a system based on a rational targeting of the molecular lesions that drive cancer. Our results suggest that early in the drug development process, a drug should be tested on a large panel of highly characterized human cell lines to screen for genomic biomarkers that are associated with sensitivity and resistance to the drug (as answered in Table S6 in File S3). These biomarkers could be used to select patient populations likely to benefit in clinical trials for rapid proof of clinical drug efficacy. Ideally the biomarker is also driving the pathogenic process and is the target of therapeutic intervention. The EML4-ALK translocation found in 4% of NSCLC, and HER2 over-expression in breast cancer are predictors of poor prognosis in lung and breast cancer, respectively, as well as effective targets for therapy [10], [29], [34], [35]. However, in both cases only a subpopulation of patients with the aberration respond to initial therapy, only later to become refractory, further supporting the notion that co-mutations that need to be assayed concurrently may be contributing to survival, necessitating inactivation of multiple kinases to induce cell death. Together, our data indicate that early phase co-development of combinations of drugs in parallel to co-development of combinations of biomarkers may be needed to deliver maximum clinical impact in cancer.

The second question, of importance for clinical trial design, is to know whether these mutations/biomarkers are sensitizing only when they occur in the context of a specific organ. If they are, then only these histologies should be selected for the trial. There are large differences in expressed transcriptional programs between organs that lead to differentiated lineages with specialized functions. Cancer cells appear to retain a memory of the intrinsic gene expression program of their differentiated original lineage [36]. We reasoned that original lineage of a cancer cell could act as a co-event in determining sensitivity or resistance to a drug in the presence of a specific mutation. However, the results suggest that while lineage is a co-event with single mutations in predicting drug sensitivity, this is most likely due to the accumulation of particular co-mutations in specific tumor lineages. If the predictive value of co-mutations across lineages persists with further experimentation, clinical trial approach may need to move from an organ based approach to a co-mutations based approach across organ sites. These findings, reproduced here in large independent sets of cell lines, drugs and biomarkers, have the potential to impact the fields of drug development, clinical trial design, and the delivery of oncology clinical care; they indicate that we may in the near future focus our treatment decisions on the multiple cooperating aberrant targets in the patient without consideration of tissue of origin. Even if relevant co-mutations are distributed at low frequency across different cancer types, a genomic-based diagnostic strategy aimed at identifying those patients who will benefit and treating them with combinatorial molecular medicine targeted to the multiple cooperating oncogenes has the potential to deliver significantly improved clinical efficacy in various human cancers.

## Supporting Information

File S1 **Figure S1, Erlotinib drug response curve and determination of sensitive and resistant cell lines.** Plot of the rank ordered GI values for erlotinib for the cell line population (n = 141, training set). The first inflection point is determined mathematically as described in the text; cell lines to the left of the inflection point are defined as sensitive, those to the right as resistant. **Figure S2, Unsupervised hierarchical clustering of the sensitivity (GI values) of 310 cell lines to 37 targeted drugs.** Magnification of Fig. 3 with annotations. **Figure S3, Unsupervised hierarchical clustering of the sensitivity (GI values) of 310 cell lines to 23 targeted drugs from the GSK set.** To decrease noise, the same data as in Fig. 3 was clustered and visualized for 23 compounds. Note the IGF1R pathway and the PI3K/AKT/mTOR pathway subclusters. **Figure S4, Unsupervised hierarchical clustering of the sensitivity (GI values) of 141 cell lines to 14 targeted drugs from the McDermott set.** To decrease noise, the same data as in Fig. 3 was clustered and visualized for 14 compounds tested in 141 cell lines. Note the EGFR pathway subcluster. **Figure S5, Flow diagram for the analysis of interaction of mutation and lineage in predicting response to a drug. Figure S6, Hierarchical clustering of the sensitivity of 18 distinct cancer lineages to 37 targeted drugs.** 302 cancer cell lines corresponding to 18 lineages with more than 3 cell lines tested per lineage were included. For each of the 37 compounds, GI values were first median centered then normalized to account for differences in potency. Hierarchical clustering was performed on the median for each lineage and drug. Increasing sensitivity of a lineage is indicated by the increasing intensity of the green signal, and increasing resistance is indicated by the increasing intensity of the red signal. Lineages not screened with a particular compound on a minimum of 2 cell lines are indicated in gray.
(PDF)

File S2 **Figure S7, Genomic event prevalence by lineage of origin for the GSK set (n = 294).** The frequencies of the individual genomic events (mutation and/or copy number aberration) for the 262 affected genes were tabulated as a heatmap against the lineage of origin for 294 cell lines with genomic information available comprising 20 lineages with more than one cell line per lineage (292 cell lines total). The occurrence of a mutation and/or copy number aberration for one gene in one cell line was counted as one occurrence. The frequency of each genomic event is indicated by the increasing intensity of the green signal.
(XLSX)

File S3 **Tables S1–S26.**
(XLSX)
