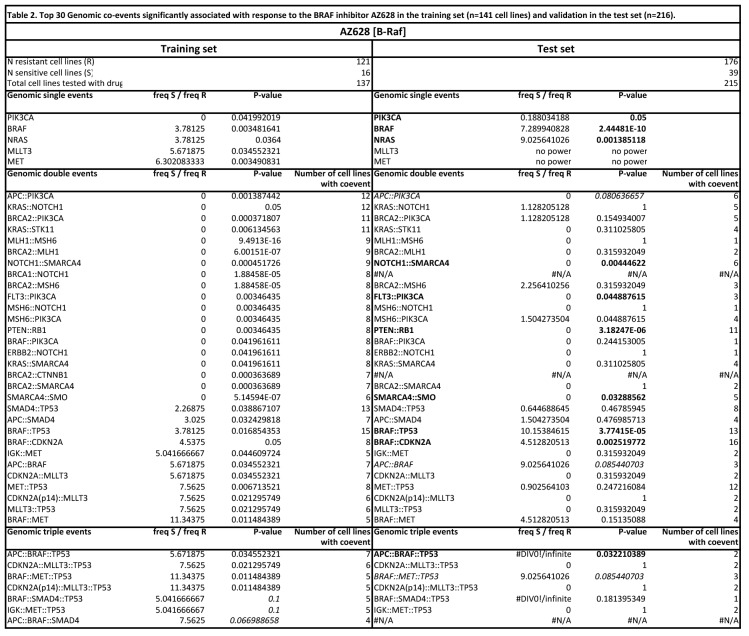# Correction: Systematic Identification of Combinatorial Drivers and Targets in Cancer Cell Lines

**DOI:** 10.1371/annotation/85d86c29-4ba6-4bf0-94f6-2977b3e1c792

**Published:** 2013-05-07

**Authors:** Adel Tabchy, Nevine Eltonsy, David E. Housman, Gordon B. Mills

There were errors in Tables 1 and 2. The correct versions of the Tables are available here:

Table 1: 

**Figure:**
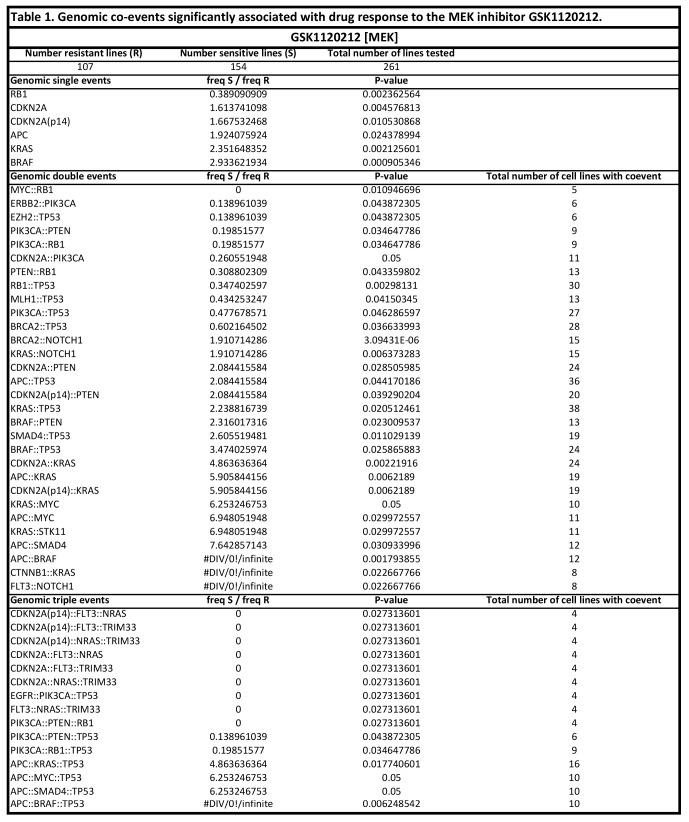


Table 2: 

**Figure:**