# Supplementary material for: Systematic Identification of Combinatorial Drivers and Targets in Cancer Cell Lines
Source: PLoS One. 2013 Apr 5;8(4):e60339. doi: 10.1371/journal.pone.0060339 (PMC3618473; doi:10.1371/journal.pone.0060339)
Supplement: File S1 — Figure S1, Erlotinib drug response curve and determination of sensitive and resistant cell lines. Plot of the rank ordered GI values for erlotinib for the cell line population (n = 141, training set). The first inflection point is determined mathematically as described in the text; cell lines to the left of the inflection point are defined as sensitive, those to the right as resistant. Figure S2, Unsupervised hierarchical clustering of the sensitivity (GI values) of 310 cell lines to 37 targeted drugs. Magnification of Fig. 3 with annotations. Figure S3, Unsupervised hierarchical clustering of the sensitivity (GI values) of 310 cell lines to 23 targeted drugs from the GSK set. To decrease noise, the same data as in Fig. 3 was clustered and visualized for 23 compounds. Note the IGF1R pathway and the PI3K/AKT/mTOR pathway subclusters. Figure S4, Unsupervised hierarchical clustering of the sensitivity (GI values) of 141 cell lines to 14 targeted drugs from the McDermott set. To decrease noise, the same data as in Fig. 3 was clustered and visualized for 14 compounds tested in 141 cell lines. Note the EGFR pathway subcluster. Figure S5, Flow diagram for the analysis of interaction of mutation and lineage in predicting response to a drug. Figure S6, Hierarchical clustering of the sensitivity of 18 distinct cancer lineages to 37 targeted drugs. 302 cancer cell lines corresponding to 18 lineages with more than 3 cell lines tested per lineage were included. For each of the 37 compounds, GI values were first median centered then normalized to account for differences in potency. Hierarchical clustering was performed on the median for each lineage and drug. Increasing sensitivity of a lineage is indicated by the increasing intensity of the green signal, and increasing resistance is indicated by the increasing intensity of the red signal. Lineages not screened with a particular compound on a minimum of 2 cell lines are indicated in gray. (PDF) [file pone.0060339.s001.pdf]

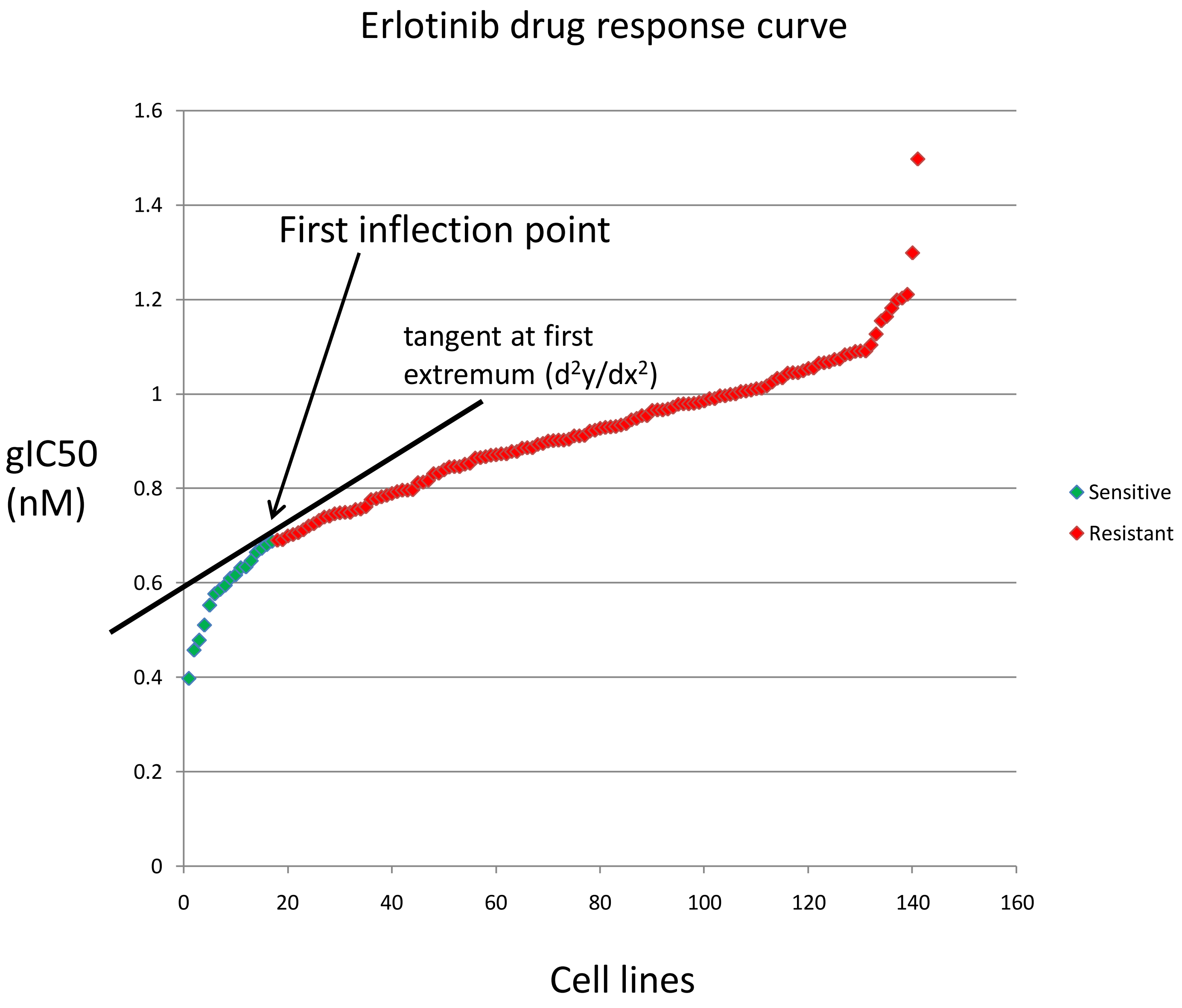
Erlotinib drug response curve
1.6
1.4
1.2
1
0.8
0.6
0.4
0.2
0
First inflection point
tangent at first extremum (d2y/dx2)
gIC50 (nM)
0
20
40
60
80
100
120
140
160
Cell lines
Sensitive
Resistant

**Figure S1.**

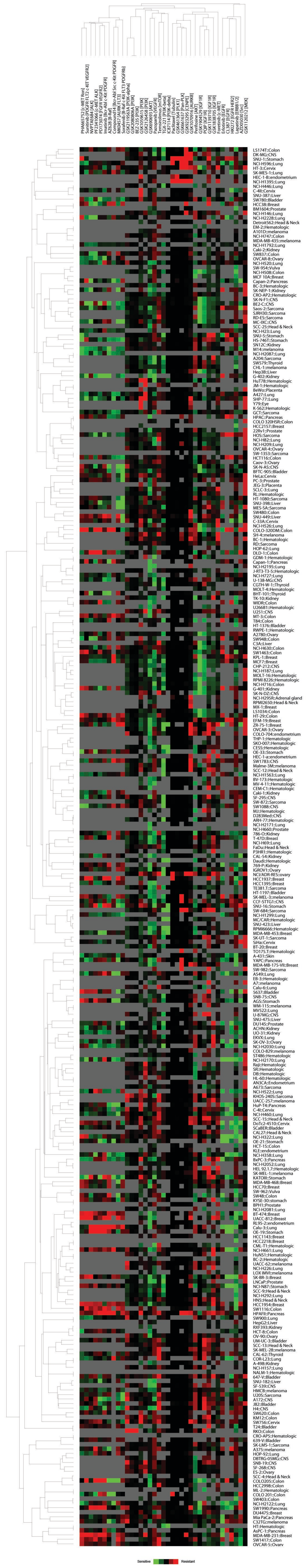


Figure S2

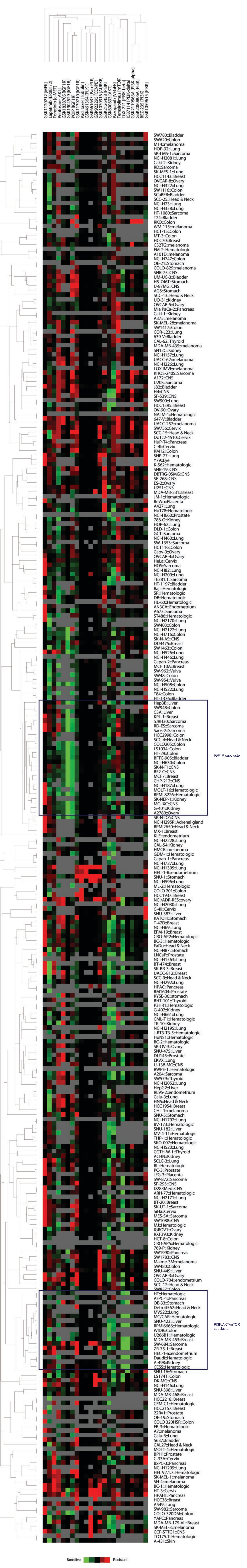
IGF1R subcluster
PI3K/AKT/mTOR subcluster
Sensitive
Resistant

Figure S3

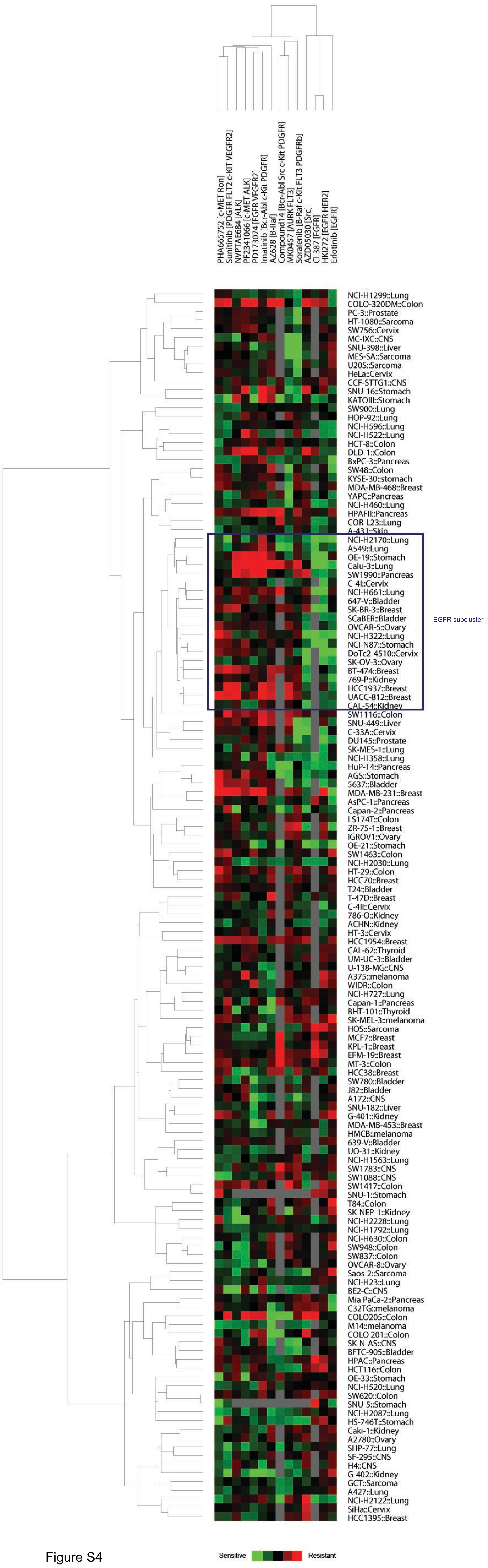
PHA665752 [c-MET Ron]
Sunitinib [PDGFR FLT2 c-KIT VEGFR2]
NVPTAE684 [ALK]
PF2341066 [c-MET ALK]
PD173074 [FGFR VEGFR2]
Imatinib [Bcr-Abl c-Kit PDGFR]
AZ628 [B-Raf]
Compound14 [Bcr-Abl Src c-Kit PDGFR]
MK0457 [AURK FLT3]
Sorafenib [B-Raf c-Kit FLT3 PDGFRb]
AZD05030 [Src]
CL387 [EGFR]
HKI272 [EGFR HER2]
Erlotinib [EGFR]
NCI-H1299::Lung
COLO-320DM::Colon
PC-3::Prostate
HT-1080::Sarcoma
SW756::Cervix
MC-IXC::CNS
SNU-398::Liver
MES-SA::Sarcoma
U20S::Sarcoma
HeLa::Cervix
CCF-STTG1::CNS
SNU-16::Stomach
KATOIII::Stomach
SW900::Lung
HOP-92::Lung
NCI-H596::Lung
NCI-H522::Lung
HCT-8::Colon
DLD-1::Colon
BxPC-3::Pancreas
SW48::Colon
KYSE-30::stomach
MDA-MB-468::Breast
YAPC::Pancreas
NCI-H460::Lung
HPAFII::Pancreas
COR-L23::Lung
A-431::Skin
NCI-H2170::Lung
A549::Lung
OE-19::Stomach
Calu-3::Lung
SW1990::Pancreas
C-4I::Cervix
NCI-H661::Lung
647-V::Bladder
SK-BR-3::Breast
SCaBER::Bladder
OVCAR-5::Ovary
NCI-H322::Lung
NCI-N87::Stomach
DoTc2-4510::Cervix
SK-OV-3::Ovary
BT-474::Breast
769-P::Kidney
HCC1937::Breast
UACC-812::Breast
CAL-54::Kidney
EGFR subcluster
SW1116::Colon
SNU-449::Liver
C-33A::Cervix
DU145::Prostate
SK-MES-1::Lung
NCI-H358::Lung
HuP-T4::Pancreas
AGS::Stomach
5637::Bladder
MDA-MB-231::Breast
AsPC-1::Pancreas
Capan-2::Pancreas
LS174T::Colon
ZR-75-1::Breast
IGROV1::Ovary
OE-21::Stomach
SW1463::Colon
NCI-H2030::Lung
HT-29::Colon
HCC70::Breast
T24::Bladder
T-47D::Breast
C-4II::Cervix
786-O::Kidney
ACHN::Kidney
HT-3::Cervix
HCC1954::Breast
CAL-62::Thyroid
UM-UC-3::Bladder
U-138-MG::CNS
A375::melanoma
WIDR::Colon
NCI-H727::Lung
Capan-1::Pancreas
BHT-101::Thyroid
SK-MEL-3::melanoma
HOS::Sarcoma
MCF7::Breast
KPL-1::Breast
EFM-19::Breast
MT-3::Colon
HCC38::Breast
SW780::Bladder
J82::Bladder
A172::CNS
SNU-182::Liver
G-401::Kidney
MDA-MB-453::Breast
HMCB::melanoma
639-V::Bladder
UO-31::Kidney
NCI-H1563::Lung
SW1783::CNS
SW1088::CNS
SW1417::Colon
SNU-1::Stomach
T84::Colon
SK-NEP-1::Kidney
NCI-H2228::Lung
NCI-H1792::Lung
NCI-H630::Colon
SW948::Colon
SW837::Colon
OVCAR-8::Ovary
Saos-2::Sarcoma
NCI-H23::Lung
BE2-C::CNS
Mia PaCa-2::Pancreas
C32TG::melanoma
COLO205::Colon
M14::melanoma
COLO 201::Colon
SK-N-AS::CNS
BFTC-905::Bladder
HPAC::Pancreas
HCT116::Colon
OE-33::Stomach
NCI-H520::Lung
SW620::Colon
SNU-5::Stomach
NCI-H2087::Lung
HS-746T::Stomach
Caki-1::Kidney
A2780::Ovary
SHP-77::Lung
SF-295::CNS
H4::CNS
G-402::Kidney
GCT::Sarcoma
A427::Lung
NCI-H2122::Lung
SiHa::Cervix
HCC1395::Breast
Sensitive
Resistant

Figure S4

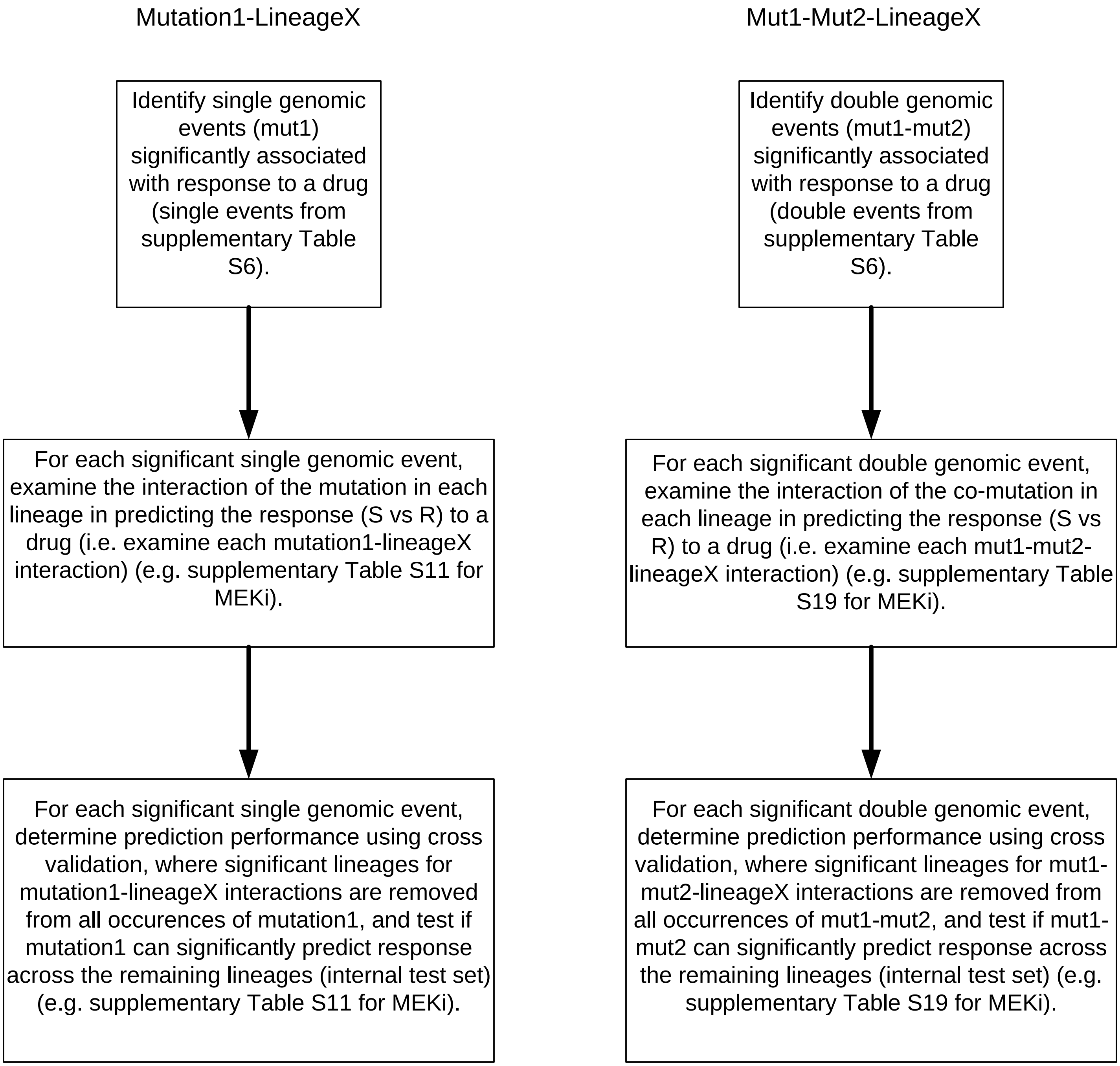
Mutation1-LineageX
Mut1-Mut2-LineageX
Identify single genomic events (mut1) significantly associated with response to a drug (single events from supplementary Table S6).
For each significant single genomic event, examine the interaction of the mutation in each lineage in predicting the response (S vs R) to a drug (i.e. examine each mutation1-lineageX interaction) (e.g. supplementary Table S11 for MEKi).
For each significant single genomic event, determine prediction performance using cross validation, where significant lineages for mutation1-lineageX interactions are removed from all occurences of mutation1, and test if mutation1 can significantly predict response across the remaining lineages (internal test set) (e.g. supplementary Table S11 for MEKi).
Identify double genomic events (mut1-mut2) significantly associated with response to a drug (double events from supplementary Table S6).
For each significant double genomic event, examine the interaction of the co-mutation in each lineage in predicting the response (S vs R) to a drug (i.e. examine each mut1-mut2-lineageX interaction) (e.g. supplementary Table S19 for MEKi).
For each significant double genomic event, determine prediction performance using cross validation, where significant lineages for mut1-mut2-lineageX interactions are removed from all occurrences of mut1-mut2, and test if mut1-mut2 can significantly predict response across the remaining lineages (internal test set) (e.g. supplementary Table S19 for MEKi).

**Figure S5.**

Figure S6

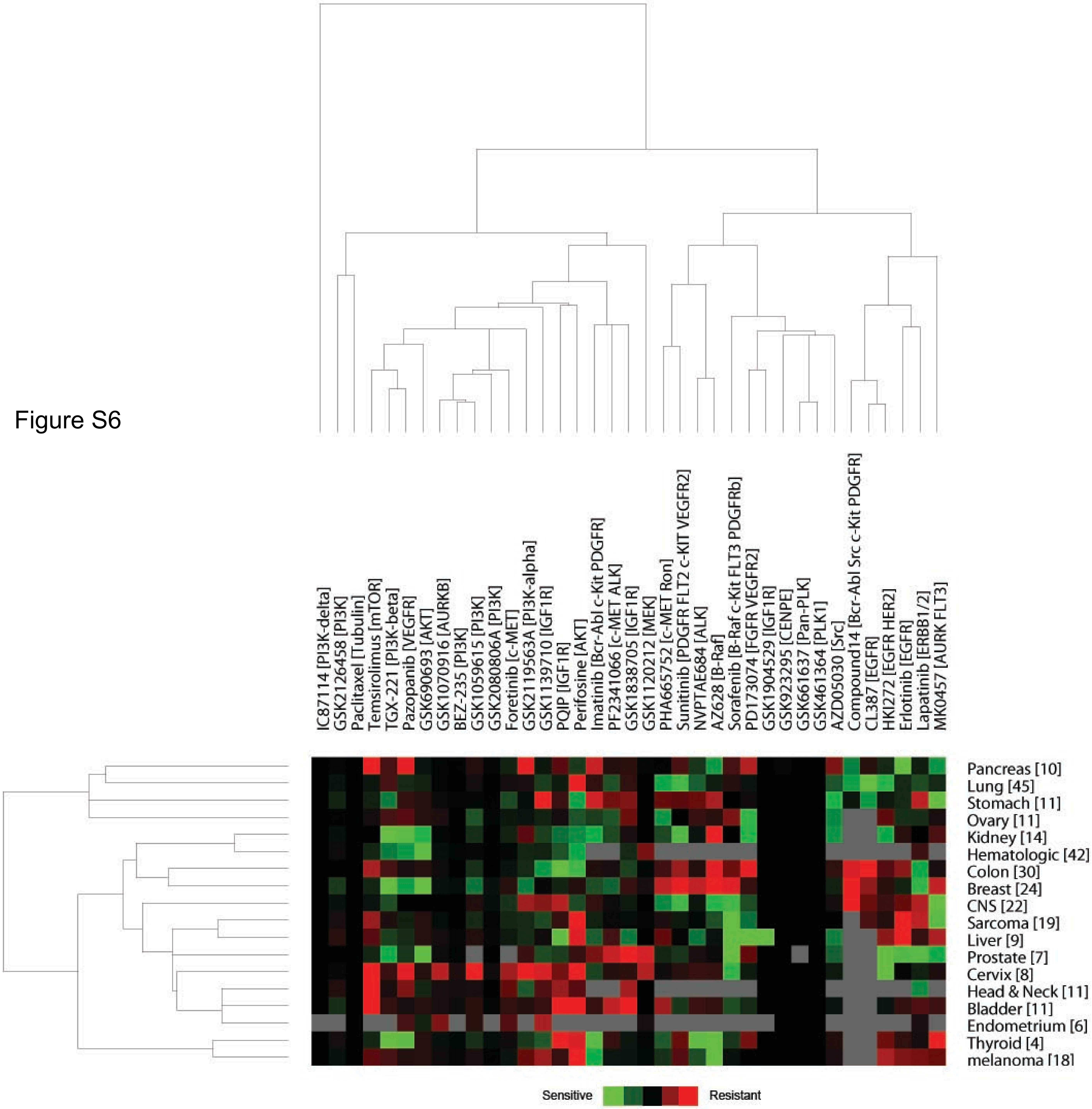
IC87114 [PI3K-delta]
GSK2126458 [PI3K]
Paclitaxel [Tubulin]
Temsirolimus [mTOR]
TGX-221 [PI3K-beta]
Pazopanib [VEGFR]
GSK690693 [AKT]
GSK1070916 [AURKB]
BEZ-235 [PI3K]
GSK1059615 [PI3K]
GSK2080806A [PI3K]
Foretinib [c-MET]
GSK2119563A [PI3K-alpha]
GSK1139710 [IGF1R]
PQIP [IGF1R]
Perifosine [AKT]
Imatinib [Bcr-Abl c-Kit PDGFR]
PF2341066 [c-MET ALK]
GSK1838705 [IGF1R]
GSK1120212 [MEK]
PHA665752 [c-MET Ron]
Sunitinib [PDGFR FLT2 c-KIT VEGFR2]
NVPTAE684 [ALK]
AZ628 [B-Raf]
Sorafenib [B-Raf c-Kit FLT3 PDGFRb]
PD173074 [FGFR VEGFR2]
GSK1904529 [IGF1R]
GSK923295 [CENPE]
GSK661637 [Pan-PLK]
GSK461364 [PLK1]
AZD05030 [Src]
Compound14 [Bcr-Abl Src c-Kit PDGFR]
CL387 [EGFR]
HKI272 [EGFR HER2]
Erlotinib [EGFR]
Lapatinib [ERBB1/2]
MK0457 [AURK FLT3]
Pancreas [10]
Lung [45]
Stomach [11]
Ovary [11]
Kidney [14]
Hematologic [42]
Colon [30]
Breast [24]
CNS [22]
Sarcoma [19]
Liver [9]
Prostate [7]
Cervix [8]
Head & Neck [11]
Bladder [11]
Endometrium [6]
Thyroid [4]
melanoma [18]
Sensitive
Resistant
